# De novo transcriptome assembly of *Pueraria montana var. lobata* and *Neustanthus phaseoloides* for the development of eSSR and SNP markers: narrowing the US origin(s) of the invasive kudzu

**DOI:** 10.1186/s12864-018-4798-3

**Published:** 2018-06-05

**Authors:** Matthew S. Haynsen, Mohammad Vatanparast, Gouri Mahadwar, Dennis Zhu, Roy Z. Moger-Reischer, Jeff J. Doyle, Keith A. Crandall, Ashley N. Egan

**Affiliations:** 1 Department of Biology, George Washington University, Washington, DC USA; 2 Computational Biology Institute, Milken Institute School of Public Health, George Washington University, Washington, DC USA; 3 Department of Botany, National Museum of Natural History, Smithsonian Institution, Washington, DC USA; 4 Present address: College of Engineering, Oregon State University, Corvallis, OR USA; 5 Present address: Department of Biology, Washington University in St. Louis, St. Louis, MO USA; 6 Present address: Department of Biology, Indiana University Bloomington, Bloomington, IN USA; 7 School of Integrated Plant Science, Plant Breeding and Genetics Section, Cornell University, Ithaca, NY USA; 8 Department of Invertebrate Zoology, National Museum of Natural History, Smithsonian Institution, Washington, DC USA

**Keywords:** *Pueraria montana var. lobata*, Kudzu, *Neustanthus phaseoloides*, Transcriptome, Invasive, Molecular markers

## Abstract

**Background:**

Kudzu, *Pueraria montana var. lobata*, is a woody vine native to Southeast Asia that has been introduced globally for cattle forage and erosion control. The vine is highly invasive in its introduced areas, including the southeastern US. Modern molecular marker resources are limited for the species, despite its importance. Transcriptomes for *P. montana* var. *lobata* and a second phaseoloid legume taxon previously ascribed to genus *Pueraria*, *Neustanthus phaseoloides,* were generated and mined for microsatellites and single nucleotide polymorphisms.

**Results:**

Roche 454 sequencing of *P. montana* var. *lobata* and *N. phaseoloides* transcriptomes produced read numbers ranging from ~ 280,000 to ~ 420,000. Trinity assemblies produced an average of 17,491 contigs with mean lengths ranging from 639 bp to 994 bp. Transcriptome completeness, according to BUSCO, ranged between 64 and 77%. After vetting for primer design, there were 1646 expressed simple sequence repeats (eSSRs) identified in *P. montana* var. *lobata* and 1459 in *N. phaseoloides.* From these eSSRs, 17 identical primer pairs, representing inter-generic phaseoloid eSSRs, were created. Additionally, 13 primer pairs specific to *P. montana* var. *lobata* were also created. From these 30 primer pairs, a final set of seven primer pairs were used on 68 individuals of *P. montana* var. *lobata* for characterization across the US, China, and Japan. The populations exhibited from 20 to 43 alleles across the seven loci. We also conducted pairwise tests for high-confidence SNP discovery from the kudzu transcriptomes we sequenced and two previously sequenced *P. montana* var. *lobata* transcriptomes. Pairwise comparisons between *P. montana* var. *lobata* ranged from 358 to 24,475 SNPs, while comparisons between *P. montana* var. *lobata* and *N. phaseoloides* ranged from 5185 to 30,143 SNPs.

**Conclusions:**

The discovered molecular markers for kudzu provide a starting point for comparative genetic studies within phaseoloid legumes. This study both adds to the current genetic resources and presents the first available genomic resources for the invasive kudzu vine. Additionally, this study is the first to provide molecular evidence to support the hypothesis of Japan as a source of US kudzu and begins to narrow the origin of US kudzu to the central Japanese island of Honshu.

**Electronic supplementary material:**

The online version of this article (10.1186/s12864-018-4798-3) contains supplementary material, which is available to authorized users.

## Background

*Pueraria montana* (Lour.) Merr. var. *lobata* (Willd.) Maesen & Almeida ex Sanjappa and Pradeep (kudzu) and *Neustanthus phaseoloides* (Roxburgh) Bentham (tropical kudzu), members of the phaseoloid clade of subfamily Papilionoideae of the Fabaceae family, are twining vines native to Southeast Asia that have been introduced globally for livestock forage, nitrogen soil enrichment, and erosion control [1]. Prior to recent molecular and taxonomic revision [2], *Neustanthus* was placed within *Pueraria*, along with ~ 17 additional species native to southeast Asia [3]. A comprehensive molecular systematic study of *Pueraria* sensu van der Maesen [4] confirmed that its species, including several legumes of economic importance, comprise a polyphyletic assemblage of separate evolutionary lineages spread across the phaseoloid clade [5].

Both kudzu and tropical kudzu share a penchant for invasiveness in their naturalized areas, the southeastern United States (US) and the pantropics, respectively. Of the two taxa, kudzu is a far greater agricultural pest and has garnered the majority of scientific inquiry. Kudzu was introduced into the US during the Centennial Exposition of 1876 in Philadelphia, Pennsylvania [6]. The vine is currently found in 30 states and is considered an agricultural pest throughout the southeastern US [7], costing millions of dollars in eradication and management measures annually [8, 9]. A major aspect that could be influencing the invasiveness and spread of kudzu are high levels of genetic variation observed across populations in the US. This could be due to multiple introductions from its native range, either of a single genetically diverse population, or from multiple genetically distinct subpopulations, potentially from different geographic regions or from more than one of the taxonomically recognized varieties of *Pueraria montana*.

Several molecular markers have been used over the past two decades to estimate the introduced and native genetic diversities of kudzu and two other *Pueraria montana* varieties: *Pueraria montana var. montana* and *Pueraria montana* var. *thomsonii* (Benth.) Wiersema ex D.B. Ward [10–15]. However, despite the ecological and economic importance of kudzu, its modern molecular marker resources are limited, lagging particularly in the characterization and development of microsatellites (SSRs) and single nucleotide polymorphisms (SNPs). Transcriptome sequencing is currently one of the most popular applications of next-generation sequencing due to its versatility, cost efficiency, and suitability for use on non-model organisms [16]. Transcriptomes are often mined for expressed simple sequence repeats (eSSRs) for marker development and genetic diversity studies. eSSRs have been shown to have greater transferability across taxa than traditional ‘anonymous’ SSRs [17, 18]. This increased transferability can be utilized in multiple ways. First, if a transcriptome is not available for the species of interest, a closely related species whose transcriptome is available can be used as a surrogate reference for microsatellite development. Second, if a researcher is studying two closely related taxa and transcriptomes are available for both, a single set of markers can be developed that work on both species to reduce costs. To this end, we have compared the transcriptomes of kudzu and tropical kudzu to identify shared eSSRs between the species in order to develop primers that can be used equally well for population genetic studies of either species, and shed light on the introduction history of the notorious invasive kudzu in the United States.

In the present study, three transcriptomes, two *P. montana* var. *lobata* and one *N. phaseoloides*, were de novo assembled and characterized. Intra- and inter-specific comparisons were made between transcriptomes and two sets of population genetic markers were identified: eSSRs and SNPs. The eSSRs were validated across Asian and North American populations of *P. montana.* var. *lobata* and used to explore population diversity and structure across native and introduced ranges. The resulting data provide genetic resources for future studies of kudzu and related genera through development of high-resolution marker sets for genetic diversity assessment and population studies.

## Results

### Transcriptome sequencing and quality control

Transcriptome sequencing produced between 279,109 and 423,426 reads per transcriptome (Table 1), with *Neustanthus phaseoloides* (hereafter CPP02) having the most reads produced. CPP02 and the greenhouse-raised kudzu (hereafter CPP27) were sequenced on the same run and were multiplexed with two other transcriptomes not reported here. While sequencing of CPP02 produced the most reads, the mean read length before cleaning was shorter than that of CPP27 (373 bp vs. 402 bp, respectively), as was the mean read length after cleaning (252 vs. 294, respectively). The tendency for shorter DNA fragments to be incorporated at the library construction phase and sequencing stage may provide an explanation for the difference in the number of raw reads produced between CPP27 and CPP02. However, following cleaning, the number of clean bases was comparable between CPP02 and CPP27, as were all other downstream metrics (Table 1). While 454 pyrosequencing was used for all three transcriptomes, the chemistries between the two CPP transcriptomes and the wild-collected kudzu (hereafter Pmnk6) transcriptome differed, with the Pmnk6 transcriptome benefiting from an improved chemistry, as seen in the increased number of raw bases, the average read length before cleaning (625 bp) and the mean clean read length (444; Table 1). These sequencing improvements translated into improved assembly statistics, such as increased mean contig length (~ 1.5× that of the CPP transcriptomes), higher N50 (1.65× CPP) and fewer singletons (Table 1). However, the improved chemistry did not lead to differences in the number of aligned reads in the assembled transcriptomes (Additional file 1).

**Table 1:** Statistics following ConDeTri cleaning and Trinity assembly

Accessions	CPP27	Pmnk6	CPP02
Number of raw reads	279,109	396,022	423,426
Number of raw bases (bp)	112,337,841	247,596,818	158,214,933
Number of clean reads	257,015	381,166	348,529
Cleaned reads / Raw reads (%)	92.1%	71.0%	82.3%
Number of clean bases (bp)	75,672,645	124,810,371	87,666,889
Mean clean read length (bp)	294	444	252
Number of aligned reads	99,248	116,524	119,452
Aligned read / Cleaned reads (%)	38.6%	41.4%	34.3%
Number of contigs	18,325	15,736	18,412
Number of bases in contigs (bp)	11,703,977	15,640,762	11,892,992
Mean contig length (bp)	639	994	646
N50 (bp)	755	1256	759
Longest contig (bp)	4335	4815	6221
Number of singletons	60,869	45,306	73,994
Singletons / Cleaned reads (%)	23.7%	16.1%	21.2%
Number of bases in singletons (bp)	17,591,281	20,431,176	18,048,611
Mean singleton length (bp)	289	451	244
Number of transcripts (contigs + singletons)	79,194	61,042	92,406

*bp* base pairs

### De novo assembly

**Fig. 1:**
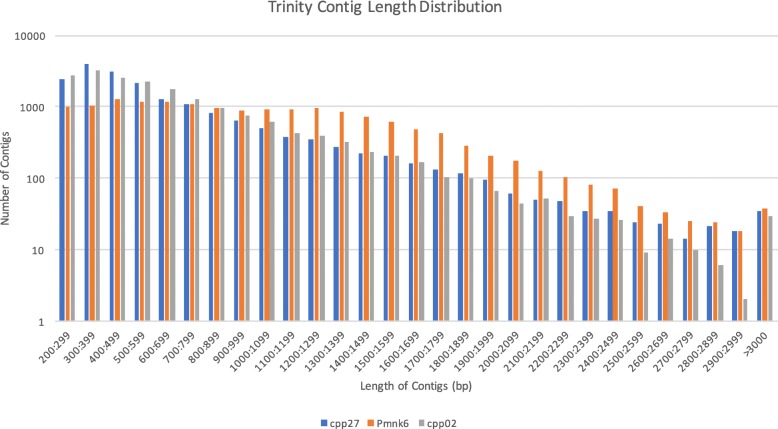
Contig length distributions of Trinity using the ConDeTri dynamic read trimmer

**Fig. 2:**
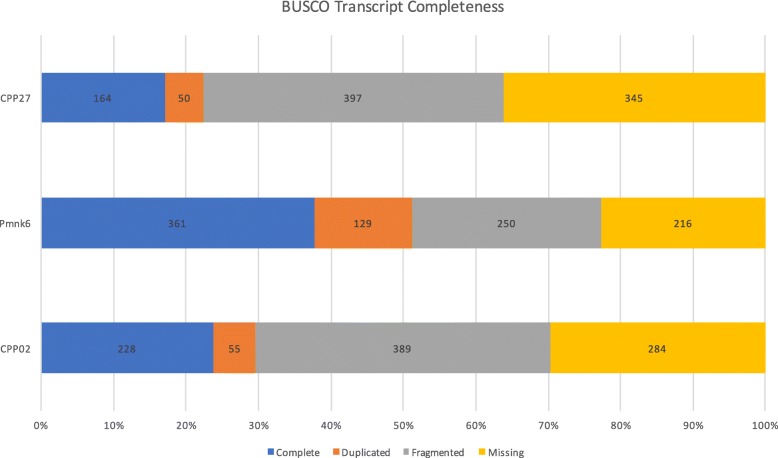
Transcriptome completeness of transcripts quantified through 956 universal single-copy orthologs using BUSCO

**Fig. 3:**
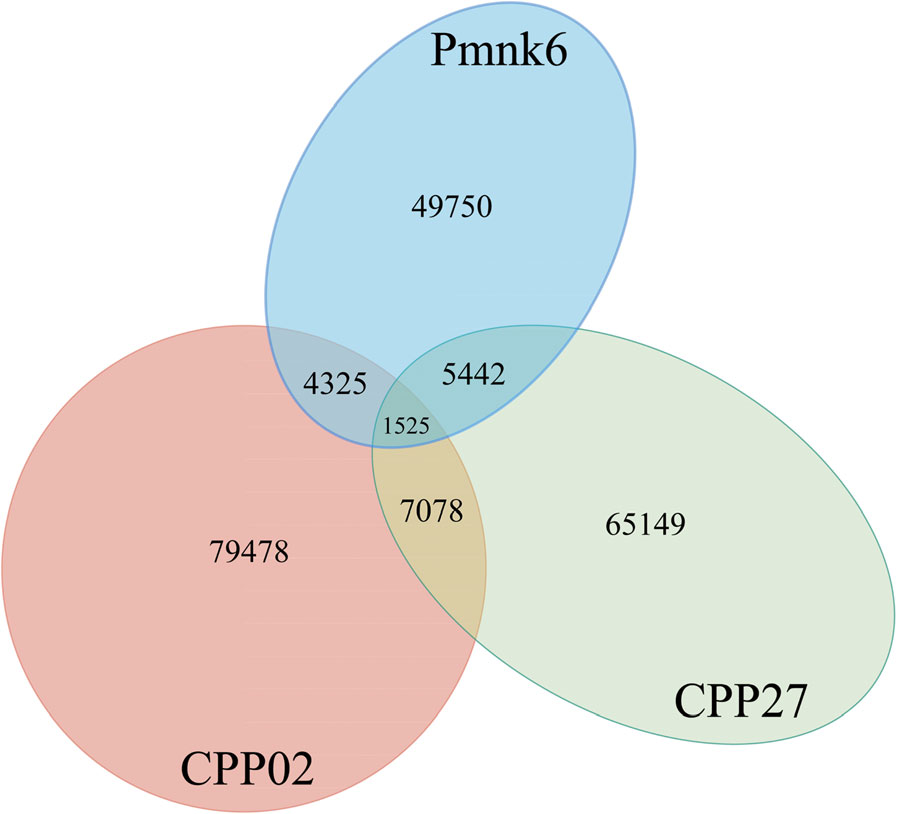
The number of unique and shared, homologous transcripts among kudzu CPP27 and Pmnk6 and tropical kudzu CPP02 transcriptome assemblies as ascertained via reciprocal best BLAST hit analyses

Trinity used an average of 38.1% of the ConDeTri cleaned reads in its assemblies and produced an average of 17,491 contigs. The mean contig lengths ranged from 639 bp to 994 bp (Table 1) and each of the accessions had contigs exceeding 3000 bp (Fig. 1). Additionally, Bowtie2 mapped ~ 68% of each accession’s contigs back to their raw reads (Additional file 1). Overall transcriptome contamination was low, with fungal contamination ranging between 2.64 and 3.53%, while prokaryote and viral contamination ranged from 0.5 to 1.32% (Additional file 2). Transcriptome completeness varied greatly, with a range of complete units from 164 to 361 and duplicate units similarly showing a > 2× difference between transcriptomes (Fig. 2). Specifically, transcriptome completeness was approximately 64, 77, and 70%, for CPP27, Pmnk6, and CPP02, respectively. The reciprocal best BLAST hits (RBH) of the transcriptomes showed that 1525 transcripts were shared among all three (Fig. 3).

### Functional annotation of transcriptomes

**Fig. 4:**
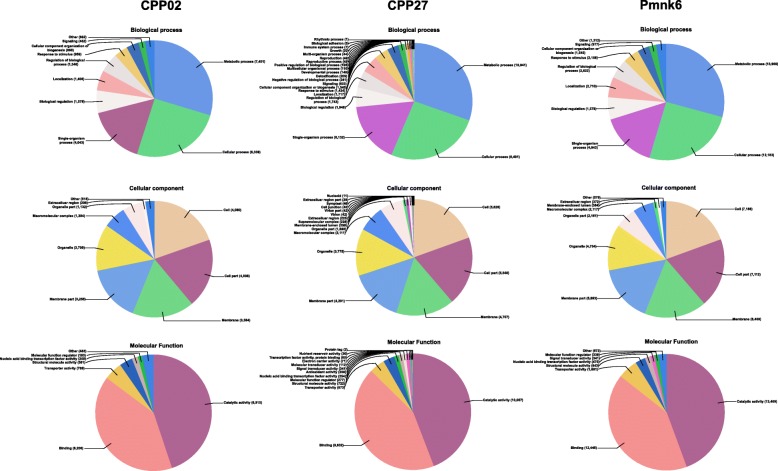
Gene ontology classifications of kudzu and tropical kudzu annotated transcripts. Numbers indicate the number of sequences associated with the particular GO term in each category

In total, we have obtained 13,230, 18,446 and 24,447 associated GO IDs for CPP02, CPP27 and Pmnk6 transcriptomes, respectively (Table 2) corresponding to the 33, 43 and 51% of original contigs in each transcriptome, while only 9.6, 17 and 36% of the singletons had associated functional protein information (GO IDs). Therefore, more than 90, 82 and 63% of singletons were discarded during the multiple searches, which is unfortunate because over 54, 56 and 66% of final annotated transcripts belong to the singletons in CPP02, CPP27, and Pmnk6, respectively (Table 2). In all three transcriptomes, the highest top hit species for the annotated proteins were *Glycine max* (L.) Merr., *G. soja* Siebold & Zucc. and *Cajanus cajan* (L.) Millsp., respectively (Additional files 3, 4, and 5). Summaries of the biological process, cellular components and molecular function categories for each transcriptome are shown in Fig. 4.

**Table 2:** Summary of gene ontology analysis

Accessions	Transcripts	Orfs	Predictions	BLAST Hits	Annotated GO IDs	ECs
CPP27	79,194	37,741	30,716	28,795	18,446	8039
	(18,325/60869)		(13,534/17182)	(12,583/16212)	(7958/10488)	
Pmnk6	61,042	50,320	42,386	39,366	24,447	6337
	(15,736/45306)		(14,821/27565)	(12,705/26661)	(8079/16368)	
CPP02	92,406	34,223	27,661	22,472	13,230	4064
	(18,412/73994)		(14,677/12984)	(10,407/12065)	(6085/7145)	

*Orfs* open reading frames, *GO* gene ontology, *ECs* enzyme codes. Parentheses: (contigs/singletons)

### SNP discovery

We conducted pairwise tests for high-confidence SNP discovery of the kudzu transcriptomes (Table 3, Additional files 6, 7, 8, 9 and 10). Our conservative assessment of SNPs reduced thousands of high-confidence SNPs to a lower number (Table 3) that are 1) one-to-one point mutations without length variants, 2) have variation frequency over 95%, and 3) have a repeat depth of 20 or more. As such, we identified 358 SNPs between the two US kudzu transcriptomes (CPP27 vs. Pmnk6), and 5185, 19,028, and 30,143 SNPs between kudzu and tropical kudzu (CPP27 vs. CPP02, Pmnk6 vs. CPP02, and CPP27/Pmnk6 vs. CPP02, respectively). The over 30,000 SNPs identified between CPP27/Pmnk6 vs. CPP02 is greater than the sum of SNPs from the individual comparisons of *P. montana* var. *lobata* to *N. phaseoloides* because the merged transcripts offer a more complete snapshot of a US kudzu transcriptome which was used as the reference for SNP detection. Lastly, we found 24,475 SNPs within kudzu from among three countries (Japan vs. Pmnk6(US)/CPP27(US)/ China). The majority of high-confidence SNPs were found within contigs rather than singletons (Table 3), which is expected given the fact that more highly expressed genes will be more likely to be represented by > 20× coverage (one of our criteria for high confidence) and are most likely to assemble into contigs. Also of note, the transition/transversion ratio varied from 1.41 to 1.73 (Table 3) with the higher ratios found between the intergeneric comparisons than the intraspecific comparisons.

**Table 3:** Single nucleotide polymorphism detection among kudzu and tropical kudzu genotypes

Comparison	HC SNPs	SNPs > 95%^a^	SNPs >20x^b^	Total SNPs^c^	Ts/Tv
Pmnk6 vs CPP27	10,417	6016	426	358	1.41
	(7494/2923)	(4125/1891)	(252/174)		
CPP02 vs CPP27	99,584	86,626	5831	5185	1.60
	(81,276/18308)	(70,638/15988)	(5091/740)		
CPP02 vs Pmnk6	220,739	174,884	21,258	19,028	1.73
	(164,118/56621)	(127,311/47573)	(19,255/2003)		
CPP02 vs Pmnk6, CPP27	314,416	248,719	33,603	30,143	1.71
	(229,163/85251)	(178,102/70617)	(29,812/3791)		
Japan vs Pmnk6, CPP27, China	494,234	79,088	27,108	24,475	1.47
	(494,234/0)	(79,088/0)	(27,108/0)		

^a^SNPs with the > 95% frequency

^b^SNPs with > 95% frequency and > 20x coverage

^c^One-to-one point mutations after exclusion of indels and length variants; HC: high confidence; parentheses: (contigs/singletons)

### eSSR discovery and characterization

The eSSR analysis of the transcripts detected 5255 and 4586 perfect eSSRs for CPP27 and CPP02, respectively. The majority (76.7 and 76.8%) of eSSRs were tri-nucleotide repeats (TNRs; Table 4). After vetting for primer design, there were 1646 potential eSSRs identified in *P. montana* var. *lobata* and 1459 in *N. phaseoloides*. Looking only at TNRs (1458 for CPP27 and 1273 for CPP02), 25 matches were found between *P. montana* var. *lobata* and *N. phaseoloides* in which either the forward or reverse primers were identical, suggesting homology. However, no sets of primer pairs (forward and reverse primers together) were found duplicated between transcriptomes. Alterations to the non-identical primer pair within the 25 matches allowed for the creation of 17 identical primer pairs between CPP27 and CPP02. These 17 shared primer pairs represent inter-generic phaseoloid eSSRs. Additionally, 13 TNR primer pairs specific to *P. montana* var. *lobata* were also selected for screening. Of the 30 total eSSR primer pairs, 21 pairs were advanced to the Culley et al. [19] protocol; of the nine primer pairs that were eliminated, four did not amplify a product, four amplified in an unexpected size range, and one displayed double banding (Additional file 11). Of the 21 primer pairs that were assessed with the Culley et al. [19] protocol, seven were discarded due to multiple banding and four for lack of amplification, whereas a further three were removed due to the presence of monomorphic alleles (Additional file 11). The final set of eSSR primer pairs identified seven polymorphic loci displaying single bands of expected sizes (Table 5).

**Table 4:** Transcriptome eSSRs

	CPP27	CPP02
Transcripts	79,194	92,406
Raw eSSRs	5255	4586
Dinucleotide	770	670
Trinucleotide	4032	3524
Tetranucleotide	180	137
Pentanucleotide	106	79
Hexanucleotide	167	176
Primered eSSRs	1646	1459
Dinucleotide	14	28
Trinucleotide	1458	1273
Tetranucleotide	62	54
Pentanucleotide	41	25
Hexanucleotide	71	79

**Table 5:** Seven eSSR primers optimized and used to assess population genetics in kudzu accessions

Locus	Sequence	Dye/Tail	SSR	Length (bp)
PP2	F: 5′-TAG GAG TGC AGC AAG CAT ATG CCG CGG ATC TTT GAA AG-3’	VIC /M13A	AAC	100–130
	R: 5’-CAA ATT GGC CCT GTC CCA AT-3’	n/a		
PP4	F: 5′-TGT AAA ACG ACG GCC AGT CAT GCC CAC GTG CTT CAT AG-3’	6FAM/M13	GCT	100–140
	R: 5’-CTC TCA GAT CCA GGC CCA AA-3’	n/a		
PP10	F: 5′-TAG GAG TGC AGC AAG CAT GGC ATG TAG ATC CAG CTA AA-3’	VIC/M13A	GGT	310–330
	R: 5′-TTG ACA GAT TTC TGA TTC TTG G-3’	n/a		
PP13	F: 5′-TAG GAG TGC AGC AAG CAT GAT TGA GCA GGC ACG AGA AC-3’	VIC/M13A	GCT	270–300
	R: 5’-CAG TAG CAG GCA TGT GTT GG-3’	n/a		
PL1	F: 5’-CAC TGC TTA GAG CGA TGC TGT AAG CGT TCG TTC GTT GG-3’	PET/M13B	CTT	400–440
	R: 5’-TCA ACC TGG TGC TCT CTG AC-3’	n/a		
PL7	F: 5′-TGT AAA ACG ACG GCC AGT AGT GGC CTT GCT CTT CTT CC-3’	6FAM/M13	CTT	80–140
	R: 5′-GTG TCA TCT CAG CAC GTT GG-3’	n/a		
PL11	F: 5′-TGT AAA ACG ACG GCC AGT TGG CAT CAT CCT TCA ACC AC-3’	6FAM/M13	ACC	300–330
	R: 5′-ATT CGG GAA TAG TGG GTG GG-3’	n/a		

*F* forward primer, *R* reverse primer. Dyes VIC: 2′-chloro-7′phenyl-1,4-dichloro-6-carboxy-fluorescein; 6FAM: 6-carboxyfluorescein; PET: chemical structure currently unpublished as proporietary to Lifetech. Tail: see Culley et al. [19] for information about M13, M13A, and M13B

### Population structure and genetic diversity of kudzu

**Fig. 5:**
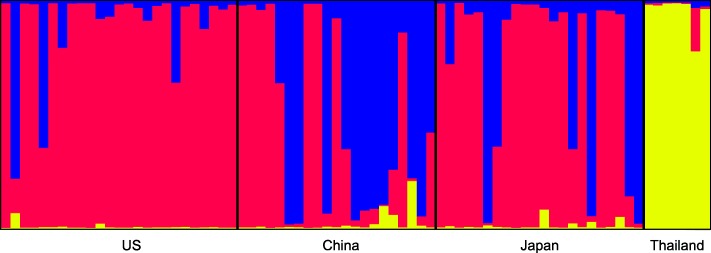
STRUCTURE diagram of 75 *P. montana* accessions across four nations (K = 3)

Three genetic units were determined to be the optimal value of K in STRUCTURE across the 75 accessions (K = 3, Fig. 5, Additional file 12). The US is primarily composed of a single genetic unit, with a couple individuals assigned to a second unit; whereas, China and Japan are more heterogeneous in their composition, yet they are still composed of the same 2 units found in the US. Thailand, however, is composed of a single genetic unit that is unique to that nation, which supports our classification of its accessions as being different varieties of *P. montana*, specifically var. *thomsonii* and var. *montana*.

The national populations exhibited from 20 to 43 alleles across a total of seven loci (Table 6), while the subpopulations exhibited from 20 to 36 total alleles (Additional file 13). China was composed of the greatest number of alleles, in particular, China 3 (southern), while Thailand was composed of the fewest number of alleles.

**Table 6:** Allelic frequency for *Pueraria* national populations

Locus	USA *N* = 25	China *N* = 21	Japan *N* = 22	Thailand *N* = 7	Mean	SD	Total
PP2	8	7	6	4	6.25	1.71	9
PP4	4	5	7	3	4.75	1.71	9
PP10	5	5	6	3	4.75	1.26	8
PP13	3	7	4	2	4.00	2.16	7
PL1	4	4	2	4	3.50	1.00	9
PL7	8	8	11	3	7.50	3.32	15
PL11	5	7	3	1	4.00	2.58	7
Mean	5.29	6.14	5.57	2.86	4.96	1.96	9.14
SD	1.98	1.46	2.99	1.07	1.42	0.80	2.73
Total	37	43	39	20	34.75	13.73	64

*N* number of accessions, *SD* standard deviation

**Fig. 6:**
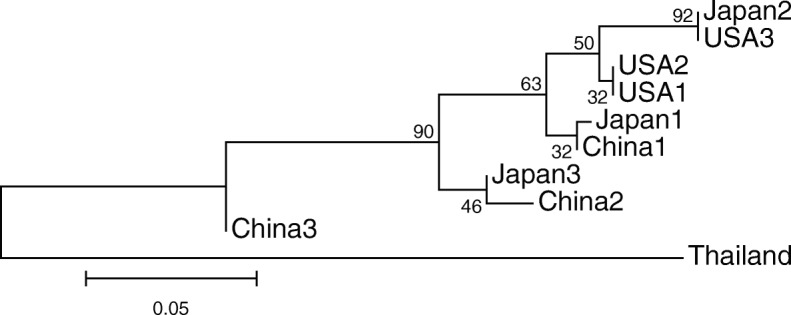
Neighbor joining distance tree based on *F*_st_ values and 10,000 bootstraps. US = United States; CN = China; JP = Japan; and TH = Thailand

After Bonferroni correction, none of the subpopulations’ observed and expected heterozygosities significantly differed (Table 7), supporting the hypothesis that all the subpopulations were in Hardy-Weinberg equilibrium when sampled. Genetic structuring as assessed by pairwise *F*_st_ showed differences among groups, particularly in Thailand and southern China (China 3; Table 8), corroborated by the structuring of genetic units shown in Fig. 5. As defined by Wright [20], Thailand showed very great genetic variation (*F*_st_ > 0.25) with respect to all other subpopulations, except China 3, with which it showed great variation (0.15 < *F*_st_ < 0.25). The rest of the comparisons resulted in little to moderate genetic variation (0 < *F*_st_ < 0.05 and 0.05 < *F*_st_ < 0.15, respectively). The neighbor-joining distance tree supports the pairwise *F*_st_ results (Fig. 6): 1) Thailand is a distantly related lineage to the nine other subpopulations representing *P. montana* var. *montana* and var. *thomsonii*; 2) the Chinese subpopulations are divided into three lineages; and 3) the US subpopulations are more genetically similar to Japan 2.

**Table 7:** Observed and expected heterozygosities for *Pueraria* subpopulations

	US 1	US 2	US 3	CN 1	CN 2	CN 3	JP 1	JP 2	JP 3	TH
# Individuals	8	10	7	5	8	8	7	8	7	7
Obs. Het.	0.717	0.552	0.472	0.611	0.378	0.632	0.396	0.506	0.656	0.594
Exp. Het.	0.643	0.503	0.547	0.648	0.589	0.763	0.579	0.572	0.661	0.583
HWE p-value	0.766	0.251	0.611	0.765	0.079	0.392	0.013	0.429	0.869	0.442

*US* United States, *CN* China, *JP* Japan, *TH* Thailand, *Obs*: Observed, *Exp* Expected, *Het* Heterozygosity, *HWE* Hardy-Weinberg Equilibrium

**Table 8:** Subpopulation pairwise *F*_st_

	US 1	US 2	US 3	CN 1	CN 2	CN 3	JP 1	JP 2	JP 3	TH
US 1	–	0.811	0.541	0.441	0.009	0.000*	0.297	0.126	0.099	0.000*
US 2	−0.023	–	0.378	0.730	0.009	0.000*	0.432	0.108	0.072	0.000*
US 3	− 0.011	− 0.008	–	0.306	0.009	0.000*	0.360	0.946	0.153	0.000*
CN 1	− 0.009	− 0.022	0.024	–	0.108	0.009	0.901	0.162	0.108	0.000*
CN 2	0.075	0.098	0.099	0.075	–	0.297	0.207	0.081	0.739	0.000*
CN 3	0.077	0.107	0.120	0.073	0.022	–	0.063	0.000*	0.324	0.000*
JP 1	0.015	−0.002	0.022	−0.035	0.051	0.064	–	0.207	0.486	0.000*
JP 2	0.016	0.025	−0.030	0.049	0.078	0.085	0.042	–	0.135	0.000*
JP 3	0.029	0.028	0.042	0.037	−0.014	0.006	0.002	0.036	–	0.000*
TH	0.315	0.370	0.377	0.330	0.310	0.244	0.347	0.352	0.322	–

Below diagonal pairwise *F*_st_ values, above diagonal *p*-values

*US* United States, *CN* China, *JP* Japan, *TH* Thailand

* = significant under Bonferroni correction (*p* < 0.001)

## Discussion

Invasive species are increasingly widening their scope across the globe, yet the genetic mechanisms underlying invasiveness or weediness remain a mystery. In the genomics era, scientists have raised a clarion call to arms to build genomic resources to study invasive species [21]. Understanding the introduction history and relative genetic diversity of invasive species is an important step to gaining a foothold on management and control, a goal requiring the development of variable molecular markers such as microsatellites or SNPs to assess genetic diversity and population structure. In this study, we have assembled and characterized multiple transcriptomes of the invasive Kudzu vine, *Pueraria montana* var. *lobata*, and for tropical kudzu, *Neustanthus phaseoloides*, a species until recently thought congeneric with kudzu [2, 5]. Kudzu is well known as an invasive species in both agricultural and natural areas due to its fast growth, clonal habit, and extensive introductions outside its native range. Tropical kudzu is also known to be invasive in its introduced ranges, but to a lesser extent. We mined our transcriptomes of these two species for molecular markers (eSSRs and SNPs), screened and validated eSSRs, and performed functional annotations of the transcriptomes, improving the genetic resources available for kudzu and tropical kudzu.

### Transcriptome characterization

Whether researching model or non-model organisms, sequencing the transcriptome of a species is a natural beginning for genome-wide resource development and study [22, 23], enabling the characterization of gene expression profiles, genetic marker discovery, and phylogenetic inference [24]. Here, we characterize the transcriptomes of two accessions of kudzu, one wild-collected (Pmnk6) and one partially inbred line propagated by the USDA agriculture research service (CPP27), as well as one of tropical kudzu (CPP02). We chose to use 454 pyrosequencing technology over Illumina due to the longer read lengths, an important consideration when dealing with potentially polyploid plants [23, 25, 26]. *Pueraria* is descendent from an ancient polyploidy event that transpired 50–60 mya near the origin of the papilionoid subfamily [27, 28], creating a duplicated genomic complement that has fractionated over time but whose signature still remains within descendent genomes. Longer reads are more likely to unambiguously assemble or align across homoeologues, duplicated genes produced via allopolyploidy [29]. Furthermore, the longer reads result in the sequencing of more full-length mRNA transcripts, an outcome that argues for including singletons (those reads that do not assemble into contigs) in the overall transcript complement. Although pyrosequencing produces fewer overall reads as compared to Illumina, its ability to produce longer transcripts is advantageous, particularly for allopolyploid species and other hybrids where avoiding the assembly of chimeric sequences is important.

The comparative results across our transcriptomes in terms of the number of transcripts discovered and the relative overlap among pairwise comparisons provides some insights into the relative impact of environment vs. shared ancestry. CPP02 had the highest number of transcripts and the highest number of unique transcripts, with Pmnk6 having the least number of transcripts, even though it presents the best transcriptome in terms of mean contig length, N50, and BUSCO results. One explanation involves the number of tissues used for sequencing. CPP02 utilized three tissues (young leaves, young shoot tips, and buds) while CPP27 used two tissues (young leaves and young shoot tips), and Pmnk6 used a single tissue (young leaves). Given this information, it makes sense that the transcriptome that was composed of the greatest number of tissues resulted in the highest number of unique transcripts due to expressional differences across tissue types. CPP02 and CPP27 shared the highest number of reciprocal best BLAST hits (RBH). However, one would expect the two kudzu accessions (CPP27 and Pmnk6) to share the greatest number of overlapping transcripts due to shared ancestry. This could also be explained by the fact that the two transcriptomes that shared the most homologous tissues resulted in the highest number of shared transcripts. Alternatively, the seeming disparity in shared best BLAST hits could be explained by the relative impacts of a shared environment, which often affects gene expression. Our two CPP transcriptomes were both grown in the same greenhouse environment at the same time and so their gene expression profiles may be expected to be more similar than those of the two *P. montana* var. *lobata* accessions, one of which was grown in the greenhouse (CPP27) and one in the wild (Pmnk6). A similar finding was discovered across transcriptomes of *Eutrema salsugineum* (Pall.) Al-Shehbaz & Warwick plants that were grown in field (uncontrolled environment) vs. cabinet (controlled environment) conditions, with the plants grown in the controlled environment sharing a higher number of expressed genes as compared to the more geographically proximate plants grown in differing environments [30].

In this study, we were able to annotate over 13,000 transcripts from kudzu and tropical kudzu (Table 1). Our transcriptomes do not provide a full gene complement due to low sequencing depth as evidence by our BUSCO results (Fig. 2). However, the level of unannotated transcripts in this study is similar to results reported from other non-model legumes, like winged bean [31], chickpea [32], and field pea [33]. The unidentified transcripts are likely due to 1) correspondence to non-coding regions or pseudogenes, 2) short length of transcripts, or 3) coding genes that have yet to be described, perhaps including species-specific “orphan” genes [34]. Catalytic activity, binding, metabolic and cellular processes were among the most highly represented groups regarding GO analysis (Fig. 4) across all three transcriptomes, as expected given that we used young tissues that are undergoing extensive metabolic activities.

### Single nucleotide polymorphism discovery

SNPs are fast becoming the marker of choice due to their ease of discovery via next generation sequencing technologies [35]. Additionally, the ease of mining SNPs from previously produced transcriptomes can provide a new use for previously published data sets that may be sitting idle in online repositories. SNPs, though less polymorphic than SSRs, may provide higher resolution assessment of genetic variation and identification of population structure [36]. We detected a near 100-fold increase in the number of SNPs detected between kudzu and tropical kudzu as compared to that detected within kudzu. SNPs discovered between kudzu and tropical kudzu may represent species level, fixed differences between these genera. Validation of these SNPs is beyond the scope of this paper; nevertheless, this list presents a significant resource for future work in genetic diversity assessment, genetic mapping, genome-wide association mapping, or evolution-based studies of invasiveness, and marks the first SNP markers discovered to date in *Pueraria* and *Neustanthus*. Use of these SNP markers across a wide population-level sampling throughout Asia would enable a robust investigation into the introduction history of kudzu within the US.

### eSSR marker discovery and validation

eSSRs are routinely developed from transcriptomic data, providing a ready source for genetic diversity assessment through cost-effective means [37]. In spite of being derived from coding DNA, which is evolutionarily conserved, eSSRs have proven a variable and valuable resource for genetic studies [18]. In our study, we detected ~ 5000 eSSRs each within kudzu and tropical kudzu. Overall, trinucleotide SSR motifs (TNRs) were the most abundant, as found consistently in other plant studies [17, 38–42]. Presumably this is because TNRs will not affect the open reading frames of coding regions [38]. We investigated the utility of 30 eSSR markers discovered in our data and optimized seven for use across kudzu. When compared to the kudzu-derived SSR markers of Hoffberg et al. [14], similarities and benefits are found. For instance, Hoffberg et al. [14] assessed their 15 genomic SSRs against 102 geographically dispersed individuals, finding that their alleles per locus ranged from 2 to 8, whereas our alleles per locus ranged from 7 to 15 (Table 6). This comparison shows twice as many alleles within a smaller sample size, approximately two-thirds the size of Hoffberg et al. [14]. One explanation for the difference in allele numbers could be attributed to the differing sampling ranges, with our individuals being collected from a greater global area. However, when Bentley and Mauricio [15] used the Hoffberg et al. [14] primers on 1747 accessions of kudzu from solely the US they identified 2–17 alleles per locus, which also represents a doubling of alleles but in a smaller sampling area. Additionally, when our observed heterozygosities are compared to the primers of Hoffberg et al. [14], they ranged from 0.372–0.726 (Table 7), while Hoffberg et al. [14] ranged from 0.0–0.9 and Bentley and Mauricio ranged from 0.004–0.741. The large difference in the heterozygosity comparisons, particularly when focusing on the low end, may be attributed to differences in sampling strategies. Bentley and Mauricio [15] report sampling kudzu within a population every few meters, suggesting that they treated a patch of kudzu as a population, whereas we sampled individuals no closer than ~ 1 km apart, and viewed a population as a regional area comprised of numerous, non-connected patches. With the abilities to grow over 12 in. per day and root at the nodes, a kudzu patch may likely represent only one or a few genets [43]. Therefore, the reported clonal sampling of Bentley and Mauricio [15] may be the cause of the near 0.0 observed heterozygosities and may not be indicative of the primers themselves.

### Genetic diversity of kudzu

For the past two decades, the genetic diversity of kudzu has been assessed with the various molecular markers of the corresponding era. For instance, Pappert et al. [10] used 13 allozymes across 1000 US accessions to conclude that introduced kudzu possessed considerable genetic variation with a lack of geographic structuring. Similar conclusions were subsequently reached by Jewett et al. [11] using 18 random amplified polymorphic DNA (RAPD) markers across 50 accessions from the US and China, and by Sun et al. [12] using 11 inter-simple sequence repeat (ISSR) markers across 108 accessions from the US and China. A decade later, Bentley and Mauricio [15], using 15 SSRs and one chloroplast marker across 1747 US accessions, reported that the high levels of genetic diversity result from high clonal reproduction in kudzu, as described by Ellstrand and Roose [44], Balloux et al. [45], and Halkett et al. [46]. Specifically, high levels of genetic variation are expected in clonal populations when the populations were founded by sexual propagules [44], which can be the case even if recruitment of sexual offspring into established populations is rare. This may be the case for kudzu due to its deliberate introduction by landowners into novel habitats from seed stock. Additionally, clonal populations are capable of maintaining higher genetic diversity at each locus even though they support a lower number of different genotypes [45, 46]. Our results corroborate the findings that introduced kudzu displays high levels of genetic variation throughout the US (Table 6, Additional file 13); however, we still maintain that the high genetic variation is possibly indicative of multiple introductions from across its native range.

### Population structure and introduction history of kudzu

Kudzu is said to have first been brought to the US by the Japanese who planted it as an ornamental vine outside their pavilion at the 1876 World’s Fair Centennial Exhibition in Philadelphia [47]. Later, David Fairchild, a plant explorer for the United States Department of Agriculture, noted its uses, including as forage, in Japan and brought back some seeds to plant near his home in Washington, D.C., as a trial. In the 1930’s, the US government began planting millions of seedlings across the southeastern states as a means of erosion control. Whether the US government sourced these kudzu seedlings from one or multiple native populations from Japan or elsewhere is not known.

Although there is consensus across most studies showing robust findings of high levels of genetic variation of kudzu in the US, most of the studies reported a lack of geographic patterning of genotypes, and none included wide sampling across Asia so as to enable an investigation into source populations of US introduction(s). Our results include new clues in identifying the native origins of US kudzu. The Thailand subpopulation is composed of non-*P. montana* var. *lobata* individuals. With evidence for strong genetic differentiation and zero population admixture between Thailand and other subpopulations, we can definitively rule Thailand out as a source of US kudzu introductions. It may also be possible to rule southern China out as an origin of US kudzu introductions due to pairwise comparisons with the central and southern US, which showed moderate levels of genetic variation (Table 8), as well as the distant placement of China 3 on the NJ tree (Fig. 6).

Of particular interest in the investigation of source populations for the introduction of US kudzu is the NJ tree clade composed of all the US subpopulations and Japan 2, the centrally located Japanese subpopulation (Fig. 6). With a bootstrap value of 50, these four subpopulations can be distinguished from the rest of the tree and within this clade, Japan 2 and US 3, the southern US, are paired together with a support of 92. These findings suggest that central Japan is a source of US kudzu. Its association with US 3, the southern US populations, makes sense considering that this area was where kudzu was first planted for soil erosion control and where farmers cultivated kudzu for fodder at the behest of the US government. Our study is the first to provide molecular evidence to support the hypothesis of Japan as a genetic source of US kudzu. However, a wider sampling across the native Asian range coupled with higher numbers of genetic markers would increase statistical power and confidence for testing genetic associations between introduced and native kudzu, efforts that are currently underway.

## Conclusions

This study produced critical genomic resources for the highly invasive kudzu vine by characterizing transcriptomes and producing marker databases for SNP and eSSR markers, foundational resources for understanding ecological adaptation that may enable future insights into invasiveness through gene discovery, marker-trait analyses, and further genetic diversity studies. We exemplified the utility of our marker databases by assessing the genetic diversity of native and introduced populations of kudzu using seven eSSRs. As a naturalized invasive vine that was intentionally introduced throughout millions of acres of the southeastern US, kudzu presents unique challenges for management, especially given its high genetic diversity across the US, a finding supported by our genetic diversity analyses. The origin of this genetic diversity remains a matter of speculation, however, this study has begun to refine the proposed hypothesis of single or multiple introductions from different genetic populations. This study is the first to provide molecular evidence that indicates the island of Honshu, Japan as one source of US kudzu. Our analyses suggest either a single introduction from a highly diverse source population in Japan, or more likely multiple introductions from multiple sources, potentially also from northern Japan (Island of Hokkaido) or northern China. Given the ecological and economic devastation wrought by kudzu in the United States, it is critical that we improve our understanding of the history, process, origin(s), and impacts of the U.S. kudzu invasion. We have assembled transcriptomes and mined them for eSSRs that we have provided as a resource for further genetic studies into the origin(s) and range expansions of kudzu to that end. By increasing both the sample ranges and sizes it should be possible to identify more accurately the origin of introduction and the number of introductions with the markers we have developed, efforts that are currently underway.

## Methods

### Plant material for transcriptome sequencing and population genetics

Transcriptomic work in this study incorporated plant tissues from two accessions of kudzu, *P. montana* var. *lobata*, and one accession of tropical kudzu, *N. phaseoloides* [formerly *Pueraria phaseoloides* (Roxb.) Benth.]. One kudzu accession (noted here as Pmnk6) was wild collected from Williamsburg, Virginia [voucher specimen G. Tate s.n. (WILLI) collected 8 July 2013]. Leaf tissue was collected in RNALater and preserved at − 20 **°** F prior to RNA extraction. The other two plants were grown from seed obtained from the United States Department of Agriculture (USDA) Germplasm Resources Information Network seed bank: accession PI 434246 of *P. montana* var. *lobata* (noted here as CPP27) was field collected in 1979 from the United States, locality unknown, and is maintained by the Coffeeville Plant Materials Center, Soil Conservation Service, Coffeeville, MS; accession PI 470272 of *N. phaseoloides* (noted here as CPP02) was donated in 1981 from a field collection by D.R. Bienz, 5 Jun 1981, Banjarbaru, S. Kalimantan, Indonesia. Seeds were grown to maturity in the greenhouse at Cornell University (Ithaca, NY, US) for 3 years prior to RNA extraction. For eSSR screening and population genetic studies, we sampled 75 accessions representing all three varieties of *P. montana* throughout their native and US introduced range: US (25), China (21), Japan (22) and Thailand (7) (Additional file 14). Leaf material was immediately stored in silica for desiccation. Genomic DNA was extracted from samples using Autogen robotics (Autogen Inc.) and a modified CTAB extraction protocol [48].

### RNA extraction and transcriptome sequencing

For the two accessions raised in the greenhouse, tissues were flash frozen in liquid nitrogen prior to RNA extraction. *Neustanthus phaseoloides* (CPP02) was sampled for young leaves, young shoot tips, and buds. Unfortunately, kudzu never flowered in the greenhouse, so only young shoot tips and young leaves were harvested for CPP27. For the wild collected kudzu (Pmnk6), only young leaves were harvested. RNA extraction, cDNA library construction, and transcriptome sequencing were carried out as previously described [31]. cDNA libraries from CPP27 and CPP02 were multiplexed with two other libraries not reported here across one titer plate on the Roche 454 Genome Sequencer FLX platform using Titanium chemistry at the Brigham Young University Sequencing Center (Provo, UT, US). Pmnk6 was also multiplexed with three other transcriptomes not reported here and sequenced using Roche 454 pyrosequencing, but using Roche’s next improvement on the titanium chemistry that produced reads ~ 800 bp long. The raw sequence data generated from CPP27, Pmnk6, and CPP02 were deposited at the National Center for Biotechnology Information (NCBI) Sequence Read Archive (SRA) under accession numbers SRR5925648, SRR5925647, and SRR5925649, respectively.

### De novo transcriptome assemblies

Raw reads were assessed for quality with FastQC [49] and subsequently cleaned with ConDeTri [50], a content-dependent read trimmer under the following settings: reads below 50 bp were removed, Phred high quality score thresholds (hq) were set to 25 and low quality score thresholds (lq) were set to 10; the fraction of bases per read having to exceed hq were set to 0.8 and the minimum number of high quality bases (mh) and maximum number of low quality bases (ml) within the sliding window were set to 30 and 5, respectively. Cleaned reads were de novo assembled using Trinity (v2.0.6) [51] under default parameters on two high-performance computing clusters: the Smithsonian Institution High Performance Cluster (SI/HPC) and the George Washington University Colonial One Cluster. In order to minimize redundant transcripts, a by-product of the assembly process, CD-HIT-EST was used with a threshold of 0.9 to obtain unique transcripts [52]. To evaluate the quality of the assemblies, criteria including the number of aligned reads, total number of contigs produced, mean contig length, N50, and transcript annotations were considered. RSEM [53] and Bowtie2 [54] were used to identify the number of aligned reads in the assembled transcriptomes. The KRAKEN suite was utilized in conjunction with prokaryote and fungal databases to identify potential contaminants within the transcriptomes [55]. BUSCO (v1.1b1), a pipeline used to accurately annotate core genes in eukaryotic genomes, was used to determine the completeness of the assemblies [56]. At the time of use, BUSCO utilized a plant core database of 956 single copy genes that are shared between *Arabidopsis*, *Oryza*, *Populus*, and *Vitis* [57]. Reciprocal Best BLAST Hits (RBH) between transcripts and among transcripts were performed on a local installation of Galaxy [58–60] and Toolshed [61] to characterize the number of shared, homologous transcripts recovered in each Trinity assembled transcriptomes [62, 63].

### Functional annotation of transcriptomes

We used transcripts (contigs + singletons) assembled by Trinity to annotate our transcriptomes (CPP27, CPP02, and Pmnk6). To identify candidate coding regions, we filtered sequences based on a minimum amino acid length of 100 using the TransDecoder program v2.0.1 [64] with the TransDecoder.LongOrfs command. BlastP and Pfam searches were carried out to detect open reading frames (ORFs) with similarity to known proteins and to maximize sensitivity for capturing ORFs that may have functional significance. The BlastP search was done using the Swissprot database with the E-value of 1E-5 and Pfam search was done using HMMER [65] and the Pfam database [66]. Output files from the BlastP and Pfam searches were used to ensure that peptides with BLAST or domain hits were retained by running the TransDecoder.Predict command. The peptide sequences from the final candidate ORFs were used to run BlastP searches against the NCBI’s nonredundant (nr) database with the E-value of 1E-5 on the SI/HPC. The BLAST results were then imported into the Blast2GO program v1.9.3 [67] to assign Gene Ontology (GO) terms. We ran mapping, annotation and InterProScan analyses for the three transcriptomes separately.

### Single nucleotide polymorphism identification

For SNP identification among the kudzu accessions, we used the transcripts (contigs + singletons) from our CPP27, Pmnk6, and CPP02 assemblies and also incorporated two publicly available *P. montana* var. *lobata* transcriptomes, SRX480408 from China derived from two tissues [68], and DRA001736 from Japan consisting of five pooled tissues [69]. We assembled the public sequences using Trinity as described above. Multiple pairwise comparisons between transcriptomes were conducted to evaluate the distribution of SNPs between US kudzu samples (CPP27 vs. Pmnk6) and identify intergeneric SNPs between kudzu and *N. phaseoloides* (CPP27 vs. CPP02 and Pmnk6 vs. CPP02). Additionally, the two US kudzu samples were combined by concatenating the two transcript files such that the samples represent the diversity in ‘US kudzu’ and subsequently compared to tropical kudzu to further identify intergeneric SNPs (CPP27/Pmnk6 vs. CPP02). Lastly, SNPs were called via comparison of all four *P. montana* var. *lobata* transcriptomes, with the transcriptome from Japan used as reference (Japan vs. CPP27/Pmnk6/China). The Japan transcriptome was chosen as reference because it incorporated the highest number of tissues, thus putatively having the higher chance of capturing greater expressed sequence diversity within the genome. To call SNPs, GS Reference Mapper v2.9 (454 Life Sciences, Roche, US) was used under default settings. The transcriptome composed of the greatest number of tissues was used as the reference to which reads from the others were assembled against. We used only high-confidence variants (454HCDiffs, > 95%) in each comparison and further filtered these variants to those having 20× or greater coverage. To ensure the highest SNP call quality, we discarded any SNPs where 1) the reference or variant involved one or more N’s or 2) the reference or variant allele was a single nucleotide insertion or deletion or did not include a point mutation in the length variant [70].

### Expressed simple sequence repeat (eSSR) loci discovery, screening and characterization

The ConDeTri cleaned, Trinity assembled, and redundancy-vetted transcripts of CPP27 and CPP02 were mined for di-, tri-, tetra-, penta-, and hexanucleotide microsatellites with MSATCOMMANDER [71]. Afterwards, MSATCOMMANDER and Primer3 [72] were used to design primer pairs for each species with an expected product size ranging from 100 to 450 bp. Primer lengths were allowed to range from 18 to 22 bp, annealing temperatures were optimized at 60 °C, and GC contents were held between 30 and 70%. Developed primers for both species were then cross-compared to identify homologous primer regions, which could signify interspecies transferability. The corresponding transcripts for primers that were shared between *P. lobata* and *N. phaseoloides* were blasted against the GenBank nonredundant database using BLASTX [73] with an *E-*value of 10^− 10^ to determine the function of their associated unigenes. Pmnk6, SRX480408 [68] and DRA001736 [69] transcriptomes were not utilized for eSSR discovery because none were available at the time eSSR mining took place. Thirty potential eSSR primer pairs were chosen from those discovered here and initially screened against a subset of accessions (Additional file 11). Seventeen of the 30 primer pairs represent putatively homologous eSSRs present in both *P. montana* var. *lobata* and *N. phaseoloides* (primer pairs designated PP) while the rest are *P. montana* var. *lobata* specific (primer pairs designated PL). The method of Culley et al. [19] was used to screen, optimize and amplify eSSRs. Primer pairs were eliminated based on the Culley et al. [19] protocol if they produced superfluous primer diming between the specific and tailed primers or produced PCR products of unexpected size. Primer pairs were further eliminated if 1) primers did not amplify viable product as seen via gel electrophoresis, 2) primers amplified more bands than expected, or 3) primers were monomorphic.

Screening of primer pairs against a subset of seven accessions ultimately yielded seven primer pairs that were characterized across all 75 accessions. Primers, fluorescent dyes, and Culley method tail adaptors used for each of the seven eSSRs are listed in Table 5. Initial rounds of amplification across the entire sampling set were performed in 12 μL reactions containing 1X Biolase NH_4_ buffer, 1.0 μL primer mix, 1.2 mM MgCl_2_, 0.12 μL of 8 μM dNTPs, 0.35 U of Taq polymerase (Biolase), and 5-80 ng DNA template. PCR was performed on an Applied Biosystems 2720 thermocycler with settings of 95C for 5 min, followed by 35 cycles of 95C for 30s, 50C for 45 s, 72C for 30s, and a final 72C extension for 5 min. Annealing temperatures were adjusted between 51.5C-58C for primers PP13, PL1, PL11, and PP2. Product bands were resolved using 1.5% sodium borate gels containing GelRed stain and visualized under UV light. Accessions that failed to amplify after two or more initial attempts were subsequently attempted with an adjusted concentration of 2.38 μg MgCl_2_ per reaction. Further failed amplifications were then tried using AmpliTaq Gold using reaction mix 1X AmpliTaq buffer, 1.0uL of primer mix, 2.86 μg MgCl_2_, 1.2uL of 8 μM dNTPs, 0.375 U of AmpliTaq Gold Taq polymerase [0.075 μL of 1000 U in 200 μL], and 5-80 ng DNA template. Successful products were genotyped using an ABI3730 sequencer at the Smithsonian NMNH LAB facilities. Genotypes were called using GeneMapper (v5.0) [74].

### Examination of population structure and genetic diversity indices

**Fig. 7:**
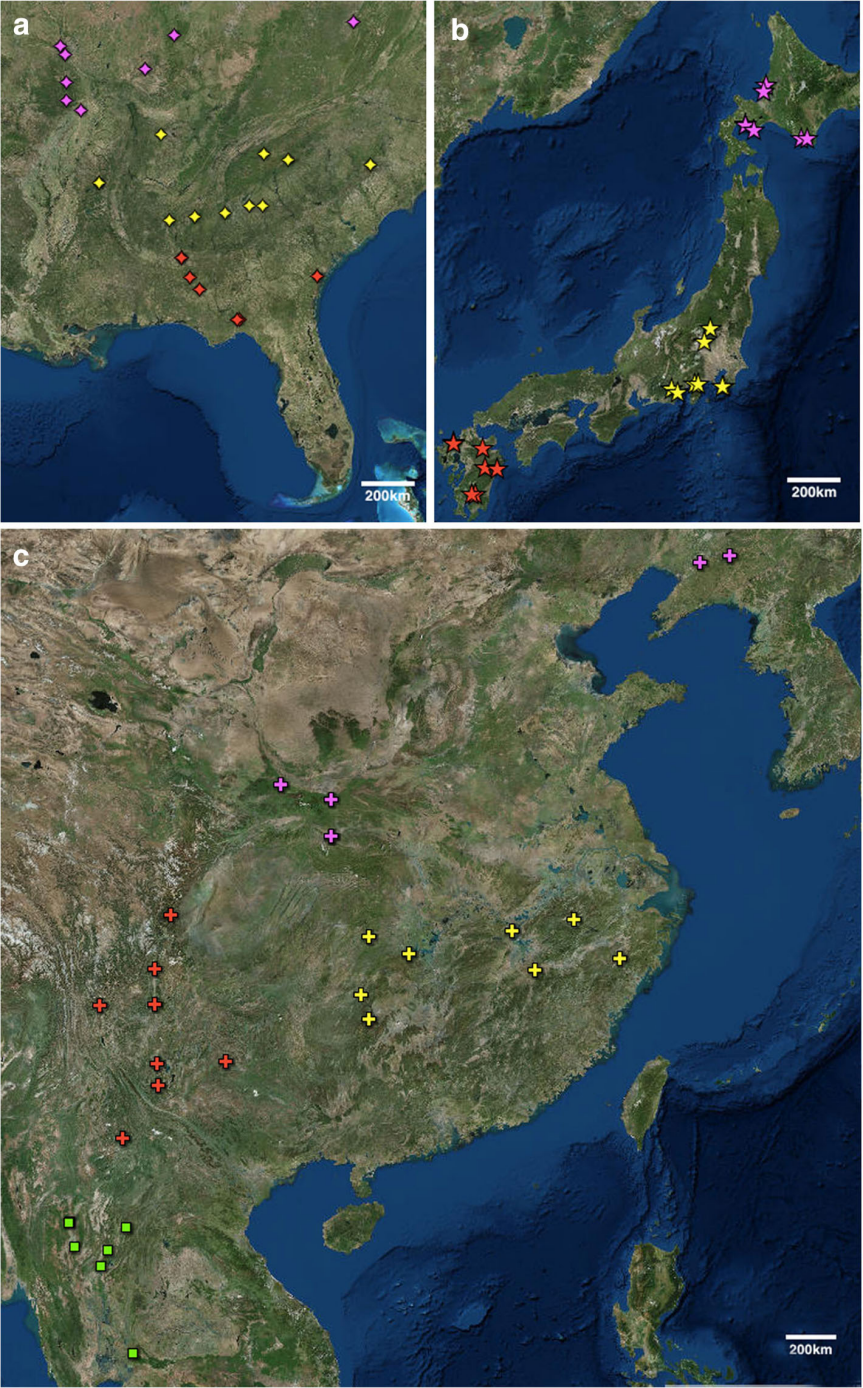
Sampling sites: (**a**) United States: US 1, US 2, US 3 (25); (**b**) Japan: JP 1, JP 2, JP 3 (22); and (**c**) China: CN 1, CN 2, CN 3 (21) and Thailand: TH (7)

Genetic population structuring was assessed with STRUCTURE v2.3.4 [75] and STRUCTURE HARVESTER v0.6.94 [76]. The length of burnin period was set to 100,000, while the number of MCMC reps after burnin was set to 900,000, resulting in a total of 1 million generations. No LOCPRIOR information was provided for the STRUCTURE runs. A job consisting of 10 iterations, evaluating Ks from 1 to 10 for the 75 *P. montana* accessions, was run and the results were uploaded to STRUCTURE Harvester for analysis. The optimal K was assessed via the Evanno et al. [77] method. Individual and population files were loaded into CLUMPP v1.1.2 [78] to address label switching and the potential for multimodality across the 10 STRUCTURE iterations. The CLUMPP program utilized the FullSearch method, the number of individuals in each population influenced weights, and the pairwise matrix similarity statistic was set to G’. All additional options remained as default settings. CLUMPP outputs for the individual and population files were visualized with DISTRUCT v1.1 [79]. Genetic diversity statistics were calculated in Arlequin v3.5.1.9 [80]. The default parameters of Arlequin were used on our 75-individual data set that was subdivided from the four sampled nations to 10 geographically defined subpopulations: US (3), China (3), Japan (3), and Thailand (1) (Fig. 7). The subpopulation designations were based primarily on geographic proximity that allowed for groupings of at-least five individuals along similar latitudinal lines; however, due to the different scales of sampling done across nations, the ranges of the latitudinal boundaries of the subpopulations differed. POPTREEW [81] was used to make a neighbor joining (NJ) distance tree with *F*_st_ distances [82] for the above listed subpopulations. Bootstrap support for the tree was calculated with 10,000 replicates.

## Additional files

Additional file 1: **Table S1.** Trinity contig reads mapped back to the raw and cleaned reads. Numbers of cleaned and raw reads mapped back to contigs via Bowtie2. (PDF 126 kb)

Additional file 2: **Table S2.** Contaminated reads as assessed by Kraken. Number (percentage) of cleaned reads annotated by Kraken as prokaryotic or fungal. (PDF 126 kb)

Additional file 3: **Figure S1.** CPP27 Top-Hit Species Distribution. Top-hit species distribution of CPP27 proteins annotated against NCBI’s non-redundant database showing the highest distribution of hits against legume species. (PDF 808 kb)

Additional file 4: **Figure S2.** Pmnk6 Top-Hit Species Distribution. Top-hit species distribution of Pmnk6 proteins annotated against NCBI’s non-redundant database showing the highest distribution of hits against legume species. (PDF 753 kb)

Additional file 5: **Figure S3.** CPP02 Top-Hit Species Distribution. Top-hit species distribution of CPP02 proteins annotated against NCBI’s non-redundant database showing the highest distribution of hits against legume species. (PDF 2607 kb)

Additional file 6: SNPs_Pmnk6_vs_CPP27. High-confidence single nucleotide polymorphisms between US kudzu accessions Pmnk6 (variant: Var) and CPP27 (reference: Ref). Accno: contig in reference; Pos: position; Nuc: nucleotide; Total Depth: number of variant reads aligned against the reference; Var Freq: frequency of variant SNP within aligned reads; # Fwd: number of forward reads with variant; # Rev.: number of reverse reads with variant; # Fwd Total: number of forward-aligned reads total; # Rev. Total: number of reverse-aligned reads total. (XLSX 578 kb)

Additional file 7: SNPs_CPP02_vs_CPP27. High-confidence single nucleotide polymorphisms between tropical kudzu CPP02 (reference: Ref) and kudzu accession CPP27 (variant: Var). Abbreviations as described for Additional file 6. (XLSX 4932 kb)

Additional file 8: SNPs_CPP02_vs_Pmnk6. High-confidence single nucleotide polymorphisms between tropical kudzu CPP02 (reference: Ref) and kudzu accession Pmnk6 (variant: Var). Abbreviations as described for Additional file 6. (XLSX 11073 kb)

Additional file 9: SNPs_CPP02_vs_Pmnk6_CPP27. High-confidence single nucleotide polymorphisms between tropical kudzu CPP02 (reference: Ref) and a composite transcriptome comprising reads from kudzu accessions CPP27 and Pmnk6 (variant: Var). Abbreviations as described for Additional file 6. (XLSX 11520 kb)

Additional file 10: SNPs_Japan_vs_Pmnk6_CPP27_China. High-confidence single nucleotide polymorphisms among kudzu accessions from Japan (reference: Ref) and reads from US kudzu (Pmnk6 and CPP27) and China (variants: Var). Abbreviations as described for Additional file 6. (XLSX 30817 kb)

Additional file 11: **Table S3.** Thirty primer pairs tested for polymorphic amplification in *Pueraria montana*. Primers labeled PP were designed from kudzu and tropical kudzu transcriptomes whereas those designated PL were designed from kudzu only. Bold primers are those used for population genetic analyses in this study. F: forward primer; R: reverse primer. (PDF 33 kb)

Additional file 12: **Figure S4.** Delta K of STRUCTURE run (K = 3). Plot of Delta K for STRUCTURE analyses from K = 2 through K = 9, with K = 3 seen as the optimal number of genetic clusters. (PDF 18 kb)

Additional file 13: **Table S4.** Allele table for Pueraria subpopulations. Number of alleles discovered for each locus within each subpopulation, with mean and standard deviation (SD) for each subpopulation and each locus. (PDF 19 kb)

Additional file 14: **Table S5.** Plant material used for eSSR validation and population genetics. Species determination, subpopulation designation (pop), country and state/province/island of origin within the United States (US), China (CN), Japan (JP) or Thailand (TH), voucher information, accession number, and geographical coordinates for each of the 75 plants used in the population genetic analyses. (PDF 34 kb)

## Abbreviations

BLAST: Basic local alignment search tool
bp: Base pair
BUSCO: Benchmarking universal single-copy orthologs
eSSR: Expressed simple sequence repeat
GO: Gene ontology
hq: High quality
lq: Low quality
mh: Minimum high quality
ml: Maximum low quality
NCBI: National Center for Biotechnology Information
nr: Nonredundant
ORF: Open reading frame
RBH: Reciprocal best hits
RIN: RNA integrity
SI/HPC: Smithsonian Institution High Performance Cluster
SNP: Single nucleotide polymorphism
SRA: Sequence read archive
SSR: Simple sequence repeat
TNR: Tri-nucleotide repeat
