# Supplementary material for: De novo transcriptome assembly of Pueraria montana var. lobata and Neustanthus phaseoloides for the development of eSSR and SNP markers: narrowing the US origin(s) of the invasive kudzu
Source: BMC Genomics. 2018 Jun 5;19:439. doi: 10.1186/s12864-018-4798-3 (PMC5989403; doi:10.1186/s12864-018-4798-3)
Supplement: Supplementary file 1 — Table S1. Trinity contig reads mapped back to the raw and cleaned reads. Numbers of cleaned and raw reads mapped back to contigs via Bowtie2. (PDF 126 kb) [file 12864_2018_4798_MOESM1_ESM.pdf]

Supplemental Table S1. Trinity contig reads mapped back to the raw and cleaned reads

	Raw			Cleaned		
**Accessions**	**CPP27**	**Pmnk6**	**CPP02**	**CPP27**	**Pmnk6**	**CPP02**
Total number of reads	279109	396022	423426	257015	281166	348529
Total number of mapped reads	189579	271541	285849	209202	244107	287296
(overall alignment rate)	(67.9%)	(68.6%)	(67.5%)	(81.4%)	(86.8%)	(82.4%)
Number of reads mapped once	129887	223624	222754	146361	198265	225010
(% of mapped reads)	(68.5%)	(82.4%)	(77.9%)	(57.0%)	(70.5%)	(64.6%)
Number of reads mapped more than once	59692	47917	63095	62841	45842	62286
(% of mapped reads)	(31.5%)	(17.6%)	(22.1%)	(24.5%)	(16.3%)	(17.9%)
Total number of unmapped reads	89530	124481	137577	47813	37059	61233
(% of unmapped reads)	(32.1%)	(31.4%)	(32.5%)	(18.6%)	(13.2%)	(17.6%)
