# Supplementary material for: De novo transcriptome assembly of Pueraria montana var. lobata and Neustanthus phaseoloides for the development of eSSR and SNP markers: narrowing the US origin(s) of the invasive kudzu
Source: BMC Genomics. 2018 Jun 5;19:439. doi: 10.1186/s12864-018-4798-3 (PMC5989403; doi:10.1186/s12864-018-4798-3)
Supplement: Supplementary file 2 — Table S2. Contaminated reads as assessed by Kraken. Number (percentage) of cleaned reads annotated by Kraken as prokaryotic or fungal. (PDF 126 kb) [file 12864_2018_4798_MOESM2_ESM.pdf]

Supplemental Table S2. Contaminated reads as assessed by Kraken

	Prokaryotes	Fungi
**CPP27**	1777 (0.50%)	6174 (2.64%)
**Pmnk6**	2209 (0.79%)	9021 (3.21%)
**CPP02**	4801 (1.32%)	12859 (3.53%)
