# Supplementary figures and images for: De novo transcriptome assembly of Pueraria montana var. lobata and Neustanthus phaseoloides for the development of eSSR and SNP markers: narrowing the US origin(s) of the invasive kudzu
Source: BMC Genomics. 2018 Jun 5;19:439. doi: 10.1186/s12864-018-4798-3 (PMC5989403; doi:10.1186/s12864-018-4798-3)

Figure S1: CPP27 Top-Hit Species Distribution

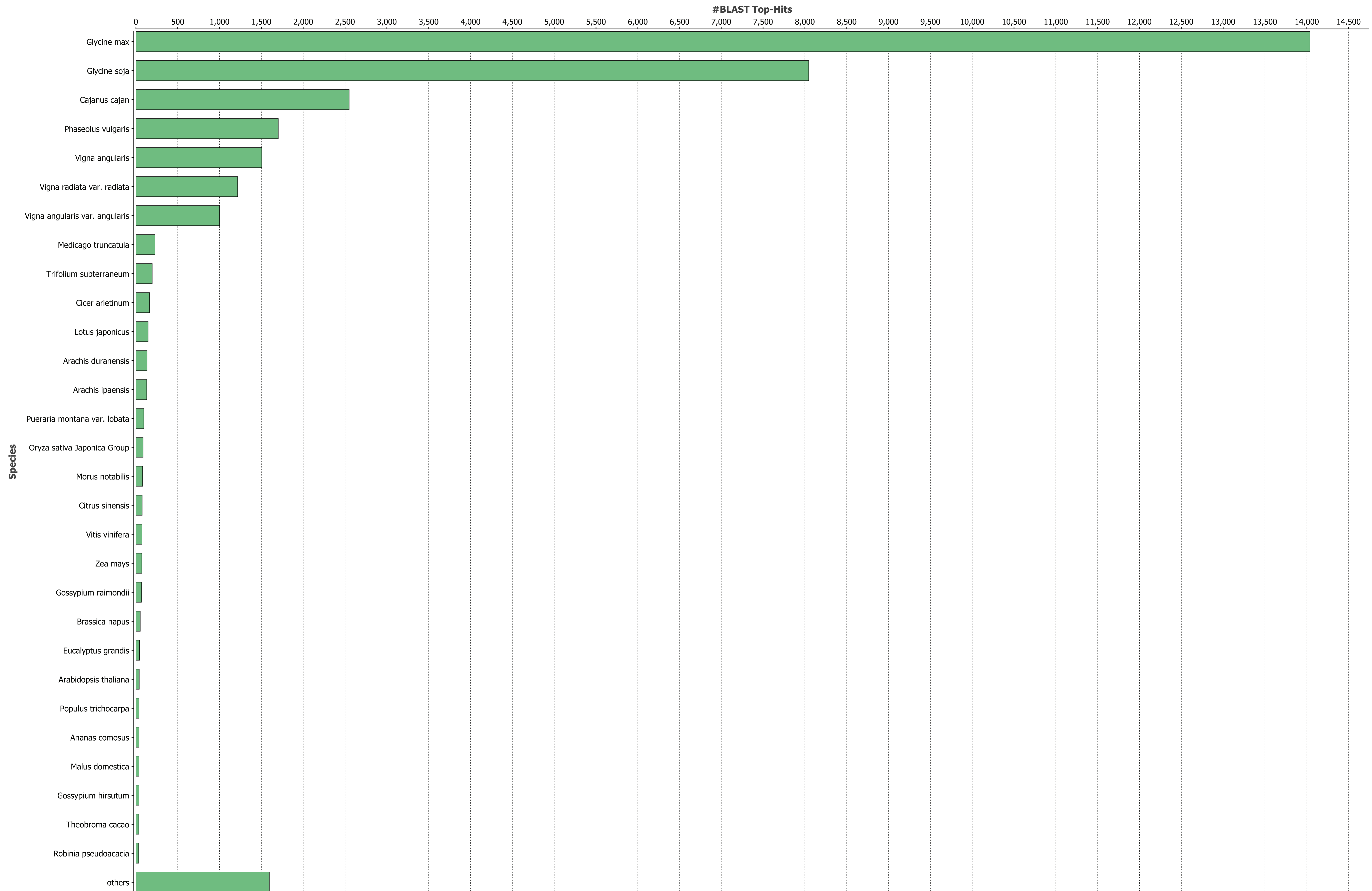

Supplement: Supplementary file 3 — Figure S1. CPP27 Top-Hit Species Distribution. Top-hit species distribution of CPP27 proteins annotated against NCBI’s non-redundant database showing the highest distribution of hits against legume species. (PDF 808 kb) [file 12864_2018_4798_MOESM3_ESM.pdf]

Figure S2: Pmnk6 Top-Hit Species Distribution

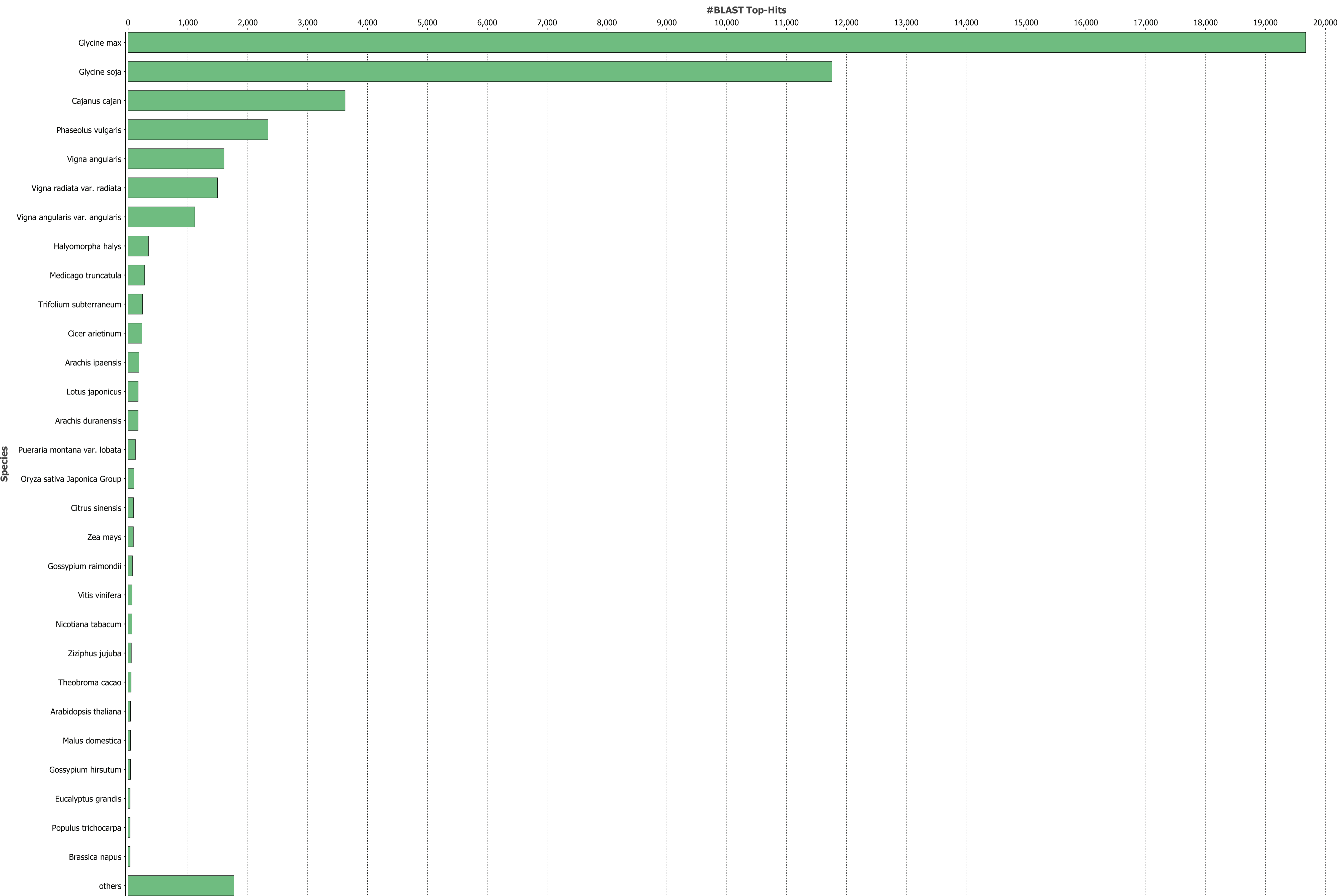

Supplement: Supplementary file 4 — Figure S2. Pmnk6 Top-Hit Species Distribution. Top-hit species distribution of Pmnk6 proteins annotated against NCBI’s non-redundant database showing the highest distribution of hits against legume species. (PDF 753 kb) [file 12864_2018_4798_MOESM4_ESM.pdf]

Figure S3: CPP02 Top-Hit Species Distribution

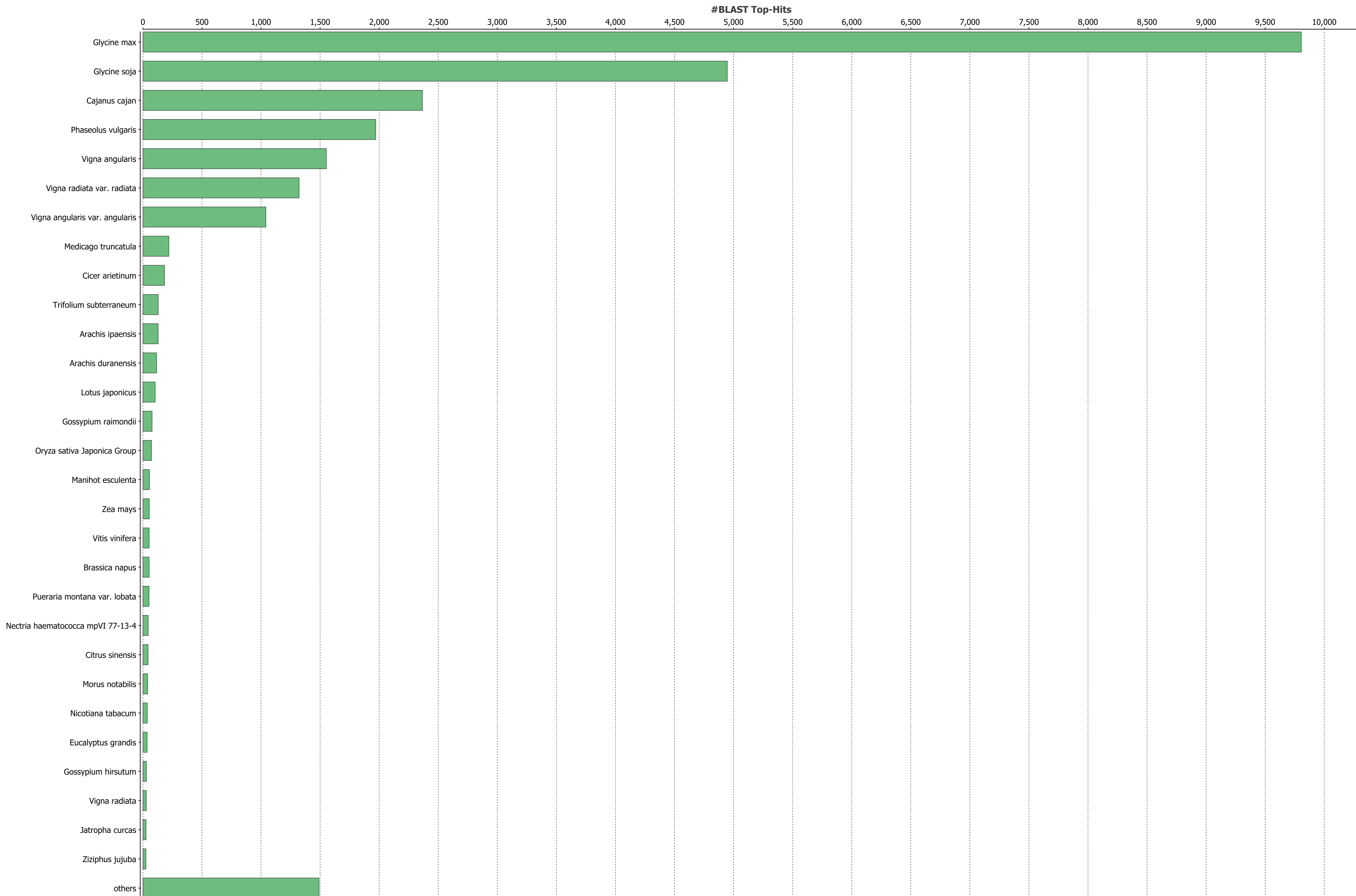

Supplement: Supplementary file 5 — Figure S3. CPP02 Top-Hit Species Distribution. Top-hit species distribution of CPP02 proteins annotated against NCBI’s non-redundant database showing the highest distribution of hits against legume species. (PDF 2607 kb) [file 12864_2018_4798_MOESM5_ESM.pdf]
