# Supplementary material for: De novo transcriptome assembly of Pueraria montana var. lobata and Neustanthus phaseoloides for the development of eSSR and SNP markers: narrowing the US origin(s) of the invasive kudzu
Source: BMC Genomics. 2018 Jun 5;19:439. doi: 10.1186/s12864-018-4798-3 (PMC5989403; doi:10.1186/s12864-018-4798-3)
Supplement: Supplementary file 11 — Table S3. Thirty primer pairs tested for polymorphic amplification in Pueraria montana. Primers labeled PP were designed from kudzu and tropical kudzu transcriptomes whereas those designated PL were designed from kudzu only. Bold primers are those used for population genetic analyses in this study. F: forward primer; R: reverse primer. (PDF 33 kb) [file 12864_2018_4798_MOESM11_ESM.pdf]

Table S3. Thirty primer pairs tested for polymorphic amplification in *Pueraria montana*

Locus		Primers	Motif	Gene or Reference	Status
PP1	F<br>R	TCGATCTCCTGGTGCGACA<br>CCCACCTTCACAGCCCTATC	$(GTT)_{4,9}$	Arginine decarboxylase	No amplification*
**PP2**	**F**<br>**R**	**ATGCCGCGGATCTTTGAAAG**<br>**CAAATTGGCCCTGTCCCAAT**	$\mathbf{(AAC)_{5,6}}$	**Calcium-dependent protein kinase**	**Polymorphic***
PP3	F<br>R	AATGTGGGAGAGCTGCAAGAT<br>TCTTGAGCAGGCTTCCGAAC	$(AAG)_5$	WEB family protein At3g02930	Monomorphic*
**PP4**	**F**<br>**R**	**CATGCCCACGTGCTTCATAG**<br>**CTCTCAGATCCAGGCCCAAA**	$\mathbf{(TGC)_5}$	**Dehydration responsive element-binding protein**	**Polymorphic***
PP5	F<br>R	TCGGCGGTTGTATTGCATTC<br>AAGGTTCACGGATCTCTCGC	$(CTT)_4$	40S ribosomal protein S30	Monomorphic*
PP6	F<br>R	TCTTCCCATGGCTACGATCG<br>AGGAGTCCGAGCTAGTGATG	$(GCT)_4$	Ras-related protein RHN1-like	No amplification*
PP7	F<br>R	TTTGCGAAGATGTGGAAGCC<br>TCAATCCTCACAGCCGTACC	$(CGG)_4$	Mitochondrial Rho GTPase 1-like	No amplification*
PP8	F<br>R	TCTGCAGGCCTAGATTTGGG<br>GGCCACATTTCCCAGAATGG	$(GTG)_5$	Chloride channel protein CLC-b-like	Monomorphic*
PP9	F<br>R	TCACCCTCAAGAAGACGAGC<br>GATTGGCAGGACTTTGTGGG	$(AGC)_4$	Plasma membrane ATPase 4-like	Wrong Band Size
**PP10**	**F**<br>**R**	**GGCATGTAGATCCAGCTAAA**<br>**TTGACAGATTTCTGATTCTTGG**	$\mathbf{(GGT)_{4,6}}$	**RNA-binding protein cabeza-like**	**Polymorphic***
PP11	F<br>R	TCAGCTGGTACAGAATCTCGA<br>GCTCACGCTCAAGGTCATTC	$(GCT)_4$	Serine/arginine-rich splicing SR45-like	No amplification
PP12	F<br>R	CAGCTGCCAAGGGATCAATG<br>TGACCCCAGATTTTTGGAAGCT	$(AGC)_4$	N-acetylglucosaminyl-transferase SEC	Multiple Bands*
**PP13**	**F**<br>**R**	**GATTGAGCAGGCACGAGAAC**<br>**CAGTAGCAGGCATGTGTTGG**	$\mathbf{(GCT)_4}$	**Signal recognition particle subunit SRP72-like**	**Polymorphic***
PP14	F<br>R	CGCCGGATTATGTCATCGAG<br>TCAACGCAGAGTACATGGGG	$(CTT)_{6,7}$	Serine/theonine-protein phosphatase PP1 isozome 4	No amplification*
PP15	F<br>R	GAACCAACCAAGGCTCCATG<br>CTGAGGTGAGTGTGGATGAA	$(AGC)_4$	SSUH2 homolog	Wrong Band Size
PP16	F<br>F	ATCACCCAAGATGGTGCCAA<br>ACAATGGTCCCTTCTGTCCC	$(GCT)_5$	Mediator of RNA polymerase II transcription subunit 15a	No amplification
PP17	R<br>F	CGGAAGAGTGCTAGTATGGG<br>ACTGGCCACTTGAGTAGGTG	$(CCG)_4$	Transcription factor MYB1R1	Multiple Bands*
**PL1**	**R**<br>**F**	**TGTAAGCGTTCGTTCGTTGG**<br>**TCAACCTGGTGCTCTCTGAC**	$\mathbf{(CTT)_7}$	**1,2-alpha-mannosidase**	**Polymorphic***
PL2	R<br>F	AGTGCTAGGGTTCGGTCAAG<br>CGCTTTCCATCCAAGTTCCC	$(CAC)_{10}$	Uncharacterized LOC100787224	Multiple Bands*
PL3	R<br>F	TCAGTTTCCGGTACAGGTGG<br>GATGCGGCAACCTTCCATC	$(GTT)_9$	U1 small nuclear ribonucleoprotein 70 kDa	Multiple Bands*
PL4	R<br>F	GTCTCGGCATGCAATCTGAC<br>ACTGTGAAAGATGTGGTGGC	$(ATC)_9$	Uncharacterized LOC100781552	Wrong Band Size
PL5	R<br>F	CATCGCCCTCATCTTCCTTC<br>TGGCCAAATTCAGGTGACAC	$(TCG)_{10}$	Uncharacterized LOC100527242	No amplification*
PL6	R<br>F	CCATGAACCTGCTTGACGAC<br>ACATGAAGAAGAAAGCGGCG	$(TGG)_5$	Uncharacterized LOC100797677	Multiple Bands*
**PL7**	**R**<br>**F**	**AGTGGCCTTGCTCTTCTTCC**<br>**GTGTCATCTCAGCACGTTGG**	$\mathbf{(TTC)_8}$	**Sphingolipid transporter spinster homolog 2**	**Polymorphic***
PL8	R<br>F	AAGGCCACTACTACCACAGC<br>CTTGCTGAATGGCCGACTTG	$(GAA)_8$	Mediator of RNA polymerase II transcription subunit 26b	Multiple Bands
PL9	R<br>F	GCACCACCATTAACCAGTCC<br>ATTCAGCGTCATCAATCGGC	$(CAA)_9$	Rop guanine nucleotide exchange factor 5-like	No amplification
PL10	R<br>F	TCACTTCATTCACAGCAGCG<br>GATGGTCCTGATCTCGTCGG	$(CCG)_6$	Heterogeneous nuclear ribonucleoprotein Q-like	No amplification
**PL11**	**F**<br>**R**	**TGGCATCATCCTTCAACCAC**<br>**ATTCGGGAATAGTGGGTGGG**	$\mathbf{(ACC)_7}$	**BRASSINAZOLE-RESISTANT 1-like**	**Polymorphic***
PL12	F<br>R	TGTTGTTGTTGCCAAGGGAG<br>TCTCAGCTATTTCCTCGGCC	$(GAT)_7$	Signal recognition particle receptor subunit alpha-like	Wrong Band Size
PL13	F<br>R	TCGATGACTACAGCTGGACG<br>TTACCCATTCTCCCACCTCC	$(GAA)_7$	Actin-related protein 3	Multiple Bands*

* primers run through the Culley et al., 2013 method; **Bold** are primers were used in eSSR analyses for this study.
