# Supplementary material for: De novo transcriptome assembly of Pueraria montana var. lobata and Neustanthus phaseoloides for the development of eSSR and SNP markers: narrowing the US origin(s) of the invasive kudzu
Source: BMC Genomics. 2018 Jun 5;19:439. doi: 10.1186/s12864-018-4798-3 (PMC5989403; doi:10.1186/s12864-018-4798-3)

Figure S4. Delta K of STRUCTURE run showing K=3 as optimal number of genetic clusters.

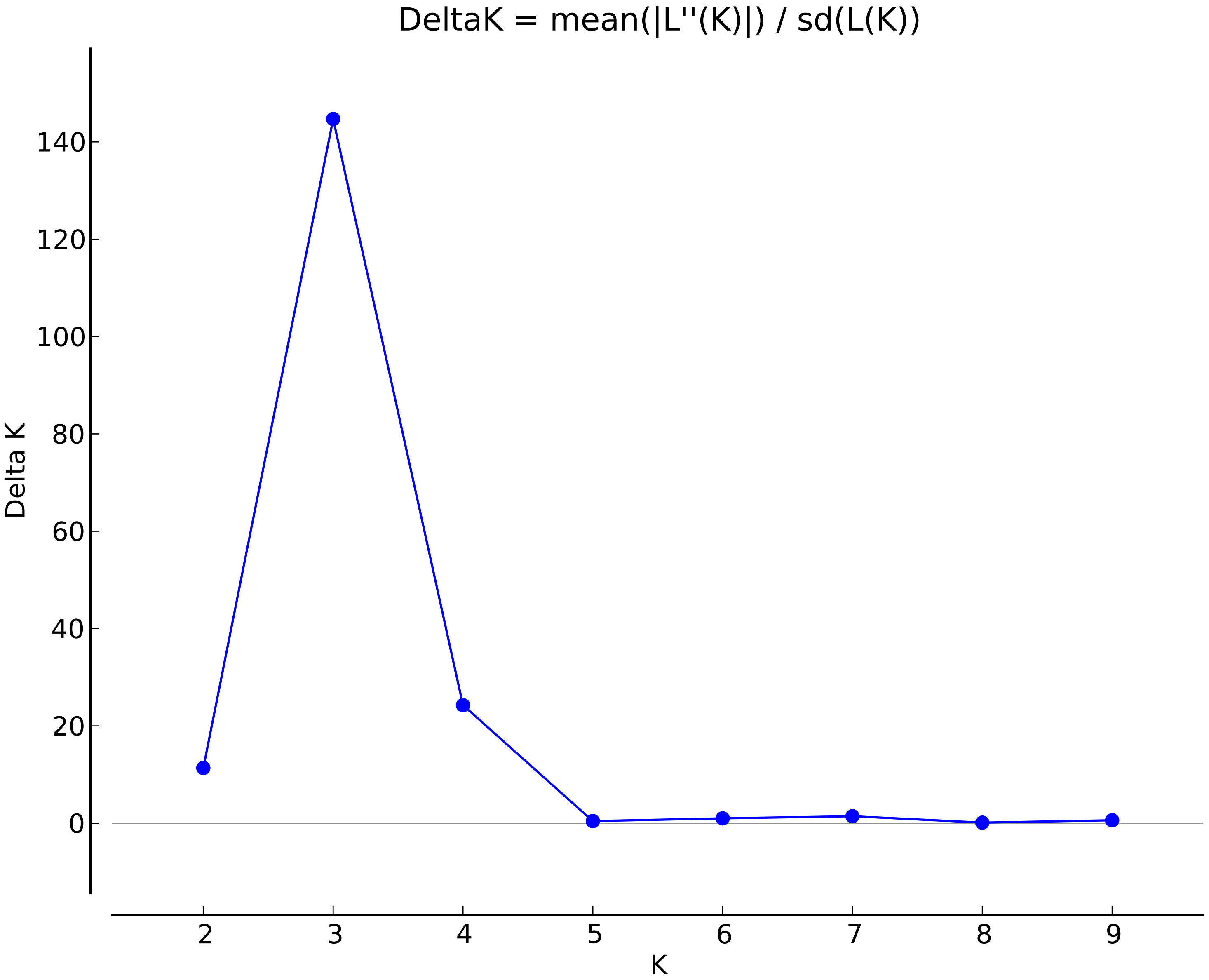

Supplement: Supplementary file 12 — Figure S4. Delta K of STRUCTURE run (K = 3). Plot of Delta K for STRUCTURE analyses from K = 2 through K = 9, with K = 3 seen as the optimal number of genetic clusters. (PDF 18 kb) [file 12864_2018_4798_MOESM12_ESM.pdf]
