# Supplementary material for: De novo transcriptome assembly of Pueraria montana var. lobata and Neustanthus phaseoloides for the development of eSSR and SNP markers: narrowing the US origin(s) of the invasive kudzu
Source: BMC Genomics. 2018 Jun 5;19:439. doi: 10.1186/s12864-018-4798-3 (PMC5989403; doi:10.1186/s12864-018-4798-3)
Supplement: Supplementary file 13 — Table S4. Allele table for Pueraria subpopulations. Number of alleles discovered for each locus within each subpopulation, with mean and standard deviation (SD) for each subpopulation and each locus. (PDF 19 kb) [file 12864_2018_4798_MOESM13_ESM.pdf]

Table S4. Allele table for *Pueraria* sub-populations

| Locus | US 1 | US 2 | US 3 | CN 1 | CN 2 | CN 3 | JP 1 | JP 2 | JP 3 | TH | Mean | SD | Total |
|---|---|---|---|---|---|---|---|---|---|---|---|---|---|
| **PP2** | 6 | 4 | 4 | 3 | 4 | 6 | 4 | 5 | 5 | 4 | 4.50 | 0.97 | 9 |
| **PP4** | 4 | 3 | 2 | 3 | 2 | 4 | 4 | 2 | 5 | 3 | 3.20 | 1.03 | 9 |
| **PP10** | 4 | 3 | 3 | 2 | 3 | 5 | 2 | 3 | 4 | 3 | 3.20 | 0.92 | 8 |
| **PP13** | 1 | 2 | 2 | 3 | 4 | 6 | 3 | 1 | 3 | 2 | 2.70 | 1.49 | 7 |
| **PL1** | 3 | 3 | 1 | 1 | 2 | 3 | 1 | 2 | 1 | 4 | 2.10 | 1.10 | 9 |
| **PL7** | 4 | 4 | 6 | 4 | 5 | 8 | 4 | 7 | 6 | 3 | 5.10 | 1.60 | 15 |
| **PL11** | 4 | 4 | 3 | 4 | 4 | 4 | 3 | 2 | 2 | 1 | 3.10 | 1.10 | 7 |
| **Mean** | 3.71 | 3.29 | 3.00 | 2.86 | 3.43 | 5.14 | 3.00 | 3.14 | 3.71 | 2.86 | | | |
| **SD** | 1.50 | 0.76 | 1.63 | 1.07 | 1.13 | 1.68 | 1.15 | 2.12 | 1.80 | 1.07 | | | |
| **Total** | 26 | 23 | 21 | 20 | 24 | 36 | 21 | 22 | 26 | 20 | | | 64 |

US = United States; CN = China; JP = Japan; TH = Thailand; SD = Standard deviation
