# Supplementary material for: De novo transcriptome assembly of Pueraria montana var. lobata and Neustanthus phaseoloides for the development of eSSR and SNP markers: narrowing the US origin(s) of the invasive kudzu
Source: BMC Genomics. 2018 Jun 5;19:439. doi: 10.1186/s12864-018-4798-3 (PMC5989403; doi:10.1186/s12864-018-4798-3)
Supplement: Supplementary file 14 — Table S5. Plant material used for eSSR validation and population genetics. Species determination, subpopulation designation (pop), country and state/province/island of origin within the United States (US), China (CN), Japan (JP) or Thailand (TH), voucher information, accession number, and geographical coordinates for each of the 75 plants used in the population genetic analyses. (PDF 34 kb) [file 12864_2018_4798_MOESM14_ESM.pdf]

Table S5. Plant Material for eSSR validation and population genetics. Photo vouchers available from corresponding author. All other vouchers held at U.S. National Herbarium (US). US: United States; CN: China; JP: Japan.

**Species**	**Pop**	**Country**	**State/Province/Island**	**Voucher**	**Accession**	**Latitude**	**Longitude**
Pueraria montana var. *lobata*	CN 1	China	Liaoning	A.N. Egan 13-001	E1840	41.0207667	123.107317
Pueraria montana var. *lobata*	CN 1	China	Liaoning	A.N. Egan 13-023	E2032	41.2402167	124.259617
Pueraria montana var. *lobata*	CN 1	China	Shaanxi	A.N. Egan 13-055	E1881	34.28175	107.04765
Pueraria montana var. *lobata*	CN 1	China	Shaanxi	A.N. Egan 13-146	E2102	32.6511667	108.96645
Pueraria montana var. *lobata*	CN 1	China	Shaanxi	A.N. Egan 13-176	E2131	33.8057667	108.9822
Pueraria montana var. *lobata*	CN 2	China	Anhui	A.N. Egan 13-314	E2222	29.88745	118.281383
Pueraria montana var. *lobata*	CN 2	China	Hunan	A.N. Egan 13-283 (photo)	E2192	29.3327833	110.425417
Pueraria montana var. *thomsonii*	CN 2	China	Hunan	A.N. Egan 13-294	E2202	28.7617333	111.958817
Pueraria montana var. *thomsonii*	CN 2	China	Hunan	A.N. Egan 13-197 (photo)	E1900	26.5411	110.428133
Pueraria montana var. *thomsonii*	CN 2	China	Hunan	A.N. Egan 13-241	E2151	27.432683	109.952917
Pueraria montana var. *lobata*	CN 2	China	Jiangxi	A.N. Egan 13-419	E2137	29.5219667	115.89175
Pueraria montana var. *thomsonii*	CN 2	China	Jiangxi	A.N. Egan 13-379	E2279	28.2224833	116.790667
Pueraria montana var. *thomsonii*	CN 2	China	Zhejiang	A.N. Egan 13-338	E2673	28.6116667	120.040083
Pueraria montana var. *lobata*	CN 3	China	Guizhou	A.N. Egan 12-284	E2977	25.08139	104.92944
Pueraria montana var. *lobata*	CN 3	China	Sichuan	A.N. Egan 12-200	E2691	30.03287	102.8138
Pueraria montana var. *lobata*	CN 3	China	Sichuan	A.N. Egan 12-209	E2919	27.05593	102.17394
Pueraria montana var. *lobata*	CN 3	China	Sichuan	A.N. Egan 12-203	E2951	28.26297	102.18277
Pueraria edulis	CN 3	China	Yunnan	A.N. Egan 12-242	E2799	24.2292	102.31336
Pueraria montana var. *lobata*	CN 3	China	Yunnan	A.N. Egan 12-227	E2773	27.00793	100.075
Pueraria montana var. *lobata*	CN 3	China	Yunnan	A.N. Egan 12-238	E2906	24.97381	102.30247
Pueraria montana var. *thomsonii*	CN 3	China	Yunnan	A.N. Egan 12-249	E2950	22.36077	100.97531
Pueraria montana var. *lobata*	JP 1	Japan	Hokkaido	A.N. Egan 13-552 (photo)	E2369	42.14917	143.00028
Pueraria montana var. *lobata*	JP 1	Japan	Hokkaido	A.N. Egan 13-556 (photo)	E2373	42.1498333	142.804667
Pueraria montana var. *lobata*	JP 1	Japan	Hokkaido	A.N. Egan 13-562 (photo)	E2378	43.5943333	141.513333
Pueraria montana var. *lobata*	JP 1	Japan	Hokkaido	A.N. Egan 13-575 (photo)	E2391	43.417	141.429833
Pueraria montana var. *lobata*	JP 1	Japan	Hokkaido	A.N. Egan 13-585 (photo)	E2401	42.40611	141.09917
Pueraria montana var. *lobata*	JP 1	Japan	Hokkaido	A.N. Egan 13-595 (photo)	E2411	42.5216667	140.7845
Pueraria montana var. *lobata*	JP 1	Japan	Hokkaido	A.N. Egan 13-596 (photo)	E2412	42.5481667	140.765333
Pueraria montana var. *lobata*	JP 2	Japan	Honshu	A.N. Egan 13-602 (photo)	E2802	35.14361	139.98611
Pueraria montana var. *lobata*	JP 2	Japan	Honshu	A.N. Egan 13-615 (photo)	E2815	35.1331667	139.951333
Pueraria montana var. *lobata*	JP 2	Japan	Honshu	A.N. Egan 13-623 (photo)	E2823	35.214	139.064333
Pueraria montana var. *lobata*	JP 2	Japan	Honshu	A.N. Egan 13-632 (photo)	E2832	35.2176667	138.925833
Pueraria montana var. *lobata*	JP 2	Japan	Honshu	A.N. Egan 13-640 (photo)	E2840	34.94639	138.33444
Pueraria montana var. *lobata*	JP 2	Japan	Honshu	A.N. Egan 13-653 (photo)	E2853	35.0653333	138.109667
Pueraria montana var. *lobata*	JP 2	Japan	Honshu	A.N. Egan 13-654 (photo)	E2854	36.4505	139.274
Pueraria montana var. *lobata*	JP 2	Japan	Honshu	A.N. Egan 13-672 (photo)	E2872	36.8575	139.5
Pueraria montana var. *lobata*	JP 3	Japan	Kyushu	A.N. Egan 13-490 (photo)	E1990	33.453667	130.1997
Pueraria montana var. *lobata*	JP 3	Japan	Kyushu	A.N. Egan 13-493 (photo)	E1993	33.432	130.203167
Pueraria montana var. *lobata*	JP 3	Japan	Kyushu	A.N. Egan 13-513 (photo)	E2334	32.6853333	131.325667
Pueraria montana var. *lobata*	JP 3	Japan	Kyushu	A.N. Egan 13-530 (photo)	E2350	32.66694	131.75
Pueraria montana var. *lobata*	JP 3	Japan	Kyushu	A.N. Egan 13-539 (photo)	E2356	31.8835	130.9765
Pueraria montana var. *lobata*	JP 3	Japan	Kyushu	A.N. Egan 13-550 (photo)	E2367	31.8741667	130.835
Pueraria montana var. *lobata*	JP 3	Japan	Kyushu	A.N. Egan 13-495 (photo)	E2682	33.27306	131.23194
Pueraria montana var. *montana*	TH	Thailand	Chiang Mai	A.N. Egan 13-803	E2519	19.3402778	98.8730556
Pueraria montana var. *montana*	TH	Thailand	Chiang Mai	A.N. Egan 13-815	E2531	19.4036111	98.9225
Pueraria montana var. *thomsonii*	TH	Thailand	Lamphun	A.N. Egan 13-784	E2031	18.4941667	99.0780556
Pueraria montana var. *montana*	TH	Thailand	Nakhon Ratchasima	A.N. Egan 13-836	E2552	14.5427778	101.351389
Pueraria montana var. *montana*	TH	Thailand	Nan	A.N. Egan 13-752	E2028	19.2038889	101.063889
Pueraria montana var. *thomsonii*	TH	Thailand	Phrae	A.N. Egan 13-746	E2026	18.3613889	100.365278
Pueraria montana var. *montana*	TH	Thailand	Uttaradit	A.N. Egan 13-736	E2468	17.7825	100.117778
Pueraria montana var. *lobata*	US 1	USA	Indiana	A.N. Egan 12-131	E1317	38.92729	-86.53724
Pueraria montana var. *lobata*	US 1	USA	Indiana	A.N. Egan 12-133	E3025	37.99589	-87.60736
Pueraria montana var. *lobata*	US 1	USA	Missouri	A.N. Egan 12-143	E1773	34.64078	-89.29037

Pueraria montana var. *lobata*	US 1	USA	Missouri	A.N. Egan 12-136	E1782	37.58647	-90.46377
Pueraria montana var. *lobata*	US 1	USA	Missouri	A.N. Egan 12-135	E1783	38.40836	-90.49232
Pueraria montana var. *lobata*	US 1	USA	Missouri	A.N. Egan 12-139	E1789	36.80336	-89.94632
Pueraria montana var. *lobata*	US 1	USA	Missouri	A.N. Egan 12-134	E1791	38.63948	-90.67005
Pueraria montana var. *lobata*	US 1	USA	Missouri	A.N. Egan 12-138	E1781	37.18877	-90.47495
Pueraria montana var. *lobata*	US 1	USA	West Virginia	A.N. Egan 12-130	E1316	39.31855	-80.03288
Pueraria montana var. *lobata*	US 2	USA	Alabama	A.N. Egan 12-59	E1019	33.53013	-86.70533
Pueraria montana var. *lobata*	US 2	USA	Alabama	A.N. Egan 12-62	E1022	33.64488	-85.82055
Pueraria montana var. *lobata*	US 2	USA	Florida	A.N. Egan 12-14	E974	30.47605	-84.18287
Pueraria montana var. *lobata*	US 2	USA	Georgia	A.N. Egan 12-79	E1039	33.75177	-84.71908
Pueraria montana var. *lobata*	US 2	USA	Georgia	A.N. Egan 12-89	E1044	33.9673	-83.82018
Pueraria montana var. *lobata*	US 2	USA	Georgia	A.N. Egan 12-103	E1058	33.94568	-83.35388
Pueraria montana var. *lobata*	US 2	USA	North Carolina	A.N. Egan 11-125	E1768	35.49912	-83.31294
Pueraria montana var. *lobata*	US 2	USA	North Carolina	A.N. Egan 11-108	E1770	35.43056	-82.24863
Pueraria montana var. *lobata*	US 2	USA	North Carolina	A.N. Egan 11-86	E1786	35.17399	-79.41421
Pueraria montana var. *lobata*	US 2	USA	Tennessee	A.N. Egan 12-146	E1771	36.20035	-87.03303
Pueraria montana var. *lobata*	US 3	USA	Alabama	A.N. Egan 12-46	E1006	32.38232	-86.29362
Pueraria montana var. *lobata*	US 3	USA	Alabama	A.N. Egan 12-55	E1015	32.38127	-86.29612
Pueraria montana var. *lobata*	US 3	USA	Alabama	A.N. Egan 12-27	E987	30.44902	-84.25945
Pueraria montana var. *lobata*	US 3	USA	Alabama	A.N. Egan 12-30	E990	31.4267	-85.61927
Pueraria montana var. *lobata*	US 3	USA	Alabama	A.N. Egan 12-35	E995	31.79347	-85.97623
Pueraria montana var. *lobata*	US 3	USA	Georgia	A.N. Egan 12-09	E969	31.81057	-81.36548
